# VPS41 recruits biosynthetic LAMP-positive vesicles through interaction with Arl8b

**DOI:** 10.1083/jcb.202405002

**Published:** 2025-02-05

**Authors:** Paolo Sanzà, Jan van der Beek, Derk Draper, Cecilia de Heus, Tineke Veenendaal, Corlinda ten Brink, Ginny G. Farías, Nalan Liv, Judith Klumperman

**Affiliations:** 1 Center for Molecular Medicine, University Medical Center Utrecht, Utrecht University, Utrecht, Netherlands; 2 Cell Biology, Neurobiology and Biophysics, Department of Biology, Faculty of Science, Utrecht University, Utrecht, Netherlands

## Abstract

Vacuolar protein sorting 41 (VPS41), a component of the homotypic fusion and protein sorting (HOPS) complex for lysosomal fusion, is essential for the trafficking of lysosomal membrane proteins via lysosome-associated membrane protein (LAMP) carriers from the trans-Golgi network (TGN) to endo/lysosomes. However, the molecular mechanisms underlying this pathway and VPS41’s role herein remain poorly understood. Here, we investigated the effects of ectopically localizing VPS41 to mitochondria on LAMP distribution. Using electron microscopy, we identified that mitochondrial-localized VPS41 recruited LAMP1- and LAMP2A-positive vesicles resembling LAMP carriers. The retention using selective hooks (RUSH) system further revealed that newly synthesized LAMPs were specifically recruited by mitochondrial VPS41, a function not shared by other HOPS subunits. Notably, we identified the small GTPase Arl8b as a critical factor for LAMP carrier trafficking. Arl8b was present on LAMP carriers and bound to the WD40 domain of VPS41, enabling their recruitment. These findings reveal a unique role of VPS41 in recruiting TGN-derived LAMP carriers and expand our understanding of VPS41–Arl8b interactions beyond endosome–lysosome fusion, providing new insights into lysosomal trafficking mechanisms.

## Teaser

This study reveals a novel role of VPS41 in cellular trafficking, showing that it recruits biosynthetic LAMP carriers in a process dependent on the small GTPase Arl8b. These findings enhance our understanding of the function of VPS41 and Arl8b beyond their role in endosome–lysosome fusion.

## Introduction

The organization of eukaryotic cells into distinct functional membrane compartments requires tightly coordinated sorting and trafficking of proteins and lipids. Vesicles shuttling between organelles are regulated by a dedicated set of proteins to safeguard the molecular identity of organelles and to allow the spatiotemporal delivery of dedicated cargos (Rothman, 2002). Lysosomes are the primary organelles for breakdown and recycling of macromolecules obtained by endocytosis or autophagy (Saftig and Klumperman, 2009; Luzio et al., 2007). Moreover, by sensing the cell nutrient status in coordination with signaling to the nucleus, lysosomes control the cell’s metabolic status (Ballabio and Bonifacino, 2020). These multiple functions are realized by the concerted action of >60 luminal acid hydrolases and >200 integral membrane proteins. Lysosomal hydrolases are principally required for degradation, whereas lysosomal membrane proteins have many different functions, such as maintaining lysosomal pH, ion composition, lysosomal membrane protection, and translocation of products over the lysosomal membrane. Lysosomal membrane proteins can also recruit cytosolic effectors required for fusion and signaling (Ballabio and Bonifacino, 2020; Eskelinen et al., 2003; Schröder et al., 2010; Callahan et al., 2009).

The targeted and timely delivery of newly synthesized lysosomal proteins is crucial to enable lysosome formation and function and is managed by various specialized pathways. The best understood pathway is mannose 6-phosphate receptor (M6PR)–dependent transport of soluble acid hydrolases. Most lysosomal hydrolases are in the Golgi complex equipped with a mannose 6-phosphate sorting signal, which in the trans-Golgi network (TGN) is recognized by cation-independent (CI) or cation-dependent (CD) M6PRs (Lobel et al., 1989; Ghosh et al., 2003). M6PRs and their bound cargo exit the TGN in clathrin-coated vesicles (Schulzf-Lohoff et al., 1985) that fuse with early endosomes (Doray et al., 2002; Puertollano et al., 2001; Waguri et al., 2003). Alternative pathways for lysosomal enzyme delivery require sortilin (Lefrancois et al., 2003; Canuel et al., 2008; Wähe et al., 2010), SEZ6L2 (Boonen et al., 2016), or lysosomal integral membrane protein-2 (Reczek et al., 2007).

Much less is known on the delivery of lysosomal membrane proteins. Lysosome-associated membrane proteins (LAMPs), including LAMP1 and LAMP2, are the most abundant membrane proteins of late endosomes and lysosomes and often used as markers to identify these organelles. Early studies showed that LAMPs can be trafficked in the same TGN-derived vesicles that carry M6PR (Höning et al., 1996) or, alternatively, travel via the constitutive pathway to the plasma membrane to reach lysosomes via subsequent endocytosis (Bonifacino and Traub, 2003; Pryor and Luzio, 2009). But recently, we identified a third pathway for delivery of LAMPs directly from the TGN to late endosomes, mediated by non–clathrin-coated vesicles that we designated “LAMP carriers” (Pols et al., 2013b). Using immuno-electron microscopy (immuno-EM), we defined LAMP carriers as vesicles measuring 70–200 nm in size, positive for LAMP1 and LAMP2, but devoid of AP1, TGN46, CI-MPR, albumin, and endocytosed BSA–gold. Fusion of LAMP carriers with late endosomes required vacuolar protein sorting 41 (VPS41) and the SNARE (soluble N-ethylmaleimide–sensitive factor attachment protein receptor) VAMP7, which were also present on the LAMP carriers. siRNA-mediated depletion of these transport proteins resulted in a significant increase in the number of LAMP carriers, which were often observed in the vicinity of endo/lysosomes (Pols et al., 2013b). In yeast, which lacks LAMPs, VPS41 is required for the alkaline phosphatase (ALP) pathway, which transports newly synthesized transmembrane proteins directly from the TGN to the vacuole (Cowles et al., 1997b; Rehling et al., 1999; Radisky et al., 1997). In *Drosophila*, we found that the *Drosophila* homologue of VPS41, Light, also regulates the lysosomal targeting of carriers transporting newly synthesized lysosomal membrane proteins. In addition to LAMP1, these carriers contained the cholesterol transporter protein NPC1 and the proton pump V0-ATPase (Swetha et al., 2011), indicating that the LAMP carrier pathway holds broad significance for transporting lysosomal membrane proteins.

Since LAMP carriers can selectively modulate the molecular composition of their target compartments, investigations into this pathway are crucial for comprehending the regulatory mechanisms of late-stage lysosome biogenesis. However, despite its clear importance, the LAMP pathway and particularly the role of VPS41 herein have largely remained unresolved. VPS41 is best known as part of the multisubunit homotypic fusion and protein sorting (HOPS) complex, which governs membrane fusions between lysosomes, late endosomes, and autophagosomes (Chou et al., 2016; van der Beek et al., 2019; Balderhaar and Ungermann, 2013; Pols et al., 2013a). The HOPS complex is closely related to the early endosomal class C core vacuole/endosome tethering (CORVET) complex, as they share four core subunits but differ in the presence of the HOPS-specific subunits VPS41 and VPS39. Deletion of VPS41 is embryonically lethal (Aoyama et al., 2012), and we and others have shown that mutations in VPS41 result in a severe neurodegenerative disease caused by a defect in HOPS-dependent endosome–lysosome fusion (Pols et al., 2013a; Steel et al., 2020; Monfrini et al., 2021; Sanderson et al., 2021; van der Welle et al., 2021). Intriguingly, VPS41 in addition to late endosomes and lysosomes was also found on TGN membranes and LAMP carriers (Pols et al., 2013b). Moreover, in yeast and secretory cells, it was suggested that VPS41 is required for vesicle formation at the TGN (Darsow et al., 2001; Asensio et al., 2013). However, recent data in yeast (Schoppe et al., 2020) and our own data in mammalian cells contradict this model, since vesicle formation proceeds in the absence of VPS41 (Pols et al., 2013b). Previously, we have shown that VPS41/HOPS depletion significantly delays endosome maturation and transport of endocytic and autophagic cargo to active lysosomes (Pols et al., 2013a; van der Beek et al., 2024). Of note, LAMP steady-state levels in lysosomes persisted, suggesting that also in case of LAMP carrier fusion, VPS41 depletion results in a delay rather than a complete blockage. Intriguingly, while VPS41 or VPS39 depletion yielded similar effects on the endosomal and autophagy pathways to lysosomes, VPS39 behaved dissimilar from VPS41 in the LAMP carrier pathway; it could not be detected on LAMP carriers (Pols et al., 2013a), and siRNA-mediated depletion did not result in the accumulation of LAMP carriers (Pols et al., 2013b). These findings raised questions regarding the role of VPS41 in LAMP carriers and whether this is in the context of the HOPS complex.

To begin to address the question how VPS41 exerts its action in the LAMP carrier pathway, we here ectopically relocalized VPS41 to mitochondria by introducing a mitochondrial targeting signal (mito-VPS41). Mitochondrial relocalization has been validated as a powerful method to investigate the in situ interactions and recruiting function of tethering proteins (Wong and Munro, 2014). Moreover, it allows study of VPS41 interactions independent of the presence of other HOPS subunits. We found that mito-VPS41 was capable of recruiting various cytoplasmic proteins, e.g., other HOPS subunits, and strikingly also LAMP carriers. Importantly, the ability to recruit LAMP carriers was unique for VPS41, as other HOPS subunits lacked this property. Furthermore, we unexpectedly found that efficient recruitment of LAMP carriers required the small GTPase Arl8b, a well-established regulator of lysosomal trafficking events (Rosa-Ferreira and Munro, 2011; Bagshaw et al., 2006; Hofmann and Munro, 2006; Khatter et al., 2015b; Pu et al., 2015). The interaction between Arl8b present on LAMP carriers and mito-VPS41 on mitochondria was critical for recruitment. Collectively, our studies reveal that mammalian VPS41 can recruit LAMP carriers, while other HOPS subunits cannot. Additionally, we unveil an unexpected role of Arl8b in the LAMP carrier pathway, expanding our understanding of VPS41–Arl8b interactions beyond conventional endosome–lysosome fusion events.

## Results

### Mito-VPS41 recruits other HOPS components via its RING domain

**Figure 1.**
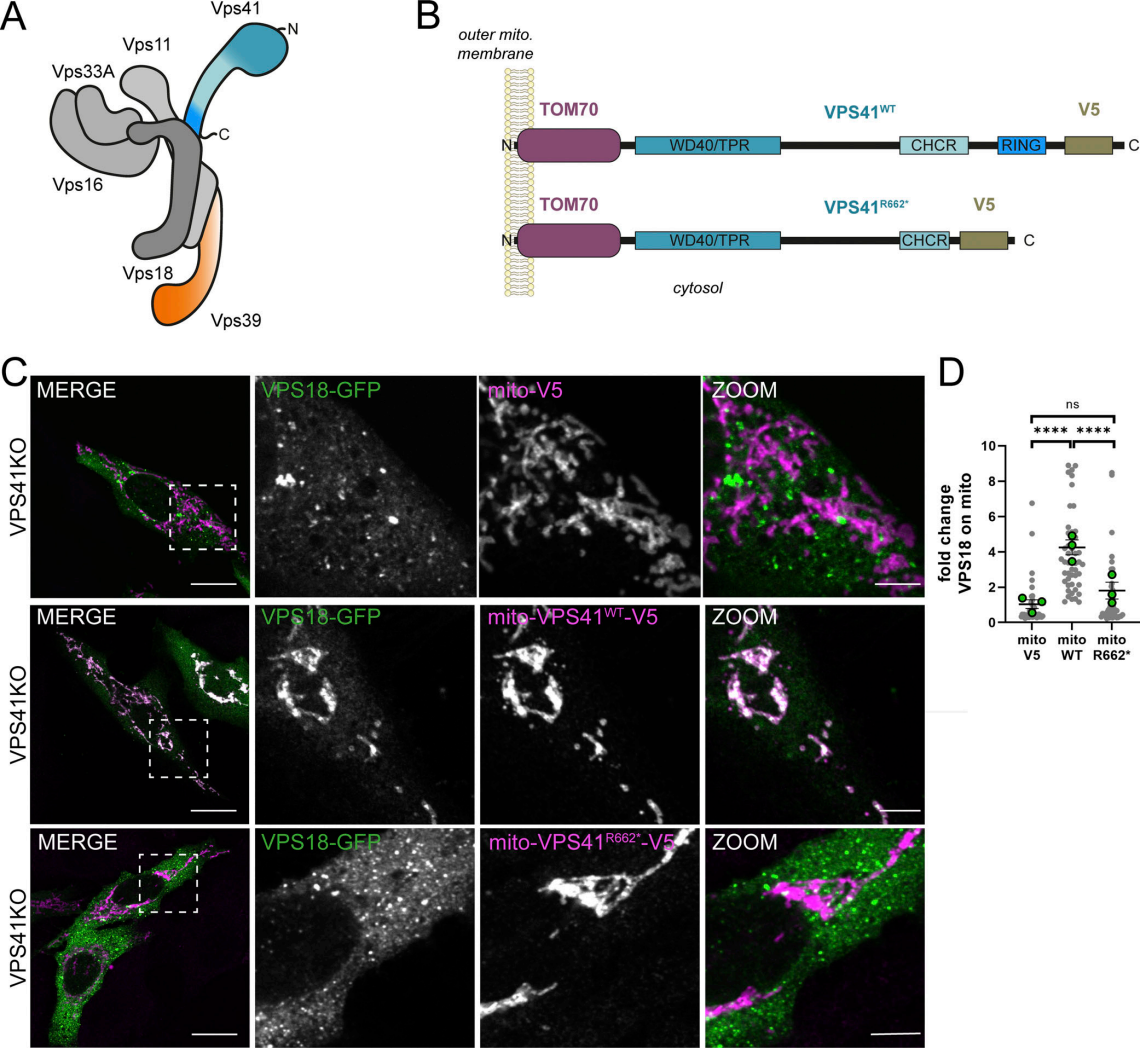
**Mito-VPS41 recruits the core HOPS subunit VPS18 via its RING domain. (A)** Schematic representation of the yeast HOPS complex (Shvarev et al., 2022). **(B)** Schematic representation of mito-VPS41^WT^-V5 and mito-VPS41^R662*^-V5 showing constituent WD40/TPR–like, CHCR, and RING domains at the C-terminal and their association with the outer mitochondrial membrane via TOM70. **(C)** Confocal images showing the distribution of VPS18-GFP co-expressed with mito-V5, mito-VPS41^WT^-V5, or mito-VPS41^R662*^-V5 (labeled for V5) in HeLa^VPS41KO^ cells. **(D)** Quantification of recruitment of VPS18-GFP to mitochondria in HeLa^VPS41KO^ cells transfected as in C, showing that the VPS41 RING domain (absent from R662*) is required for VPS18 recruitment. Fluorescence intensity of the VPS18-GFP signal within a mask of the mito-construct was normalized to the mito-V5 control condition and expressed as a fold change. Statistical significance was calculated using Tukey’s multiple comparisons test, ****P < 0.0001. *n* > 40. Data are presented as the mean ± SEM. Scale bars: 20 and 5 µm (inset). See also Fig. S1 C.

Tethering factors form the initial connection between two membranes, a prerequisite for fusion (Wong and Munro, 2014; Yu and Hughson, 2010; Orr et al., 2017; Baker et al., 2015). The HOPS tethering complex shares a core of 4 subunits (VPS18, VPS11, VPS16, and VPS33A) with the related CORVET complex required for early endosomal fusion events and is extended with the two HOPS-specific subunits VPS41 and VPS39 (Fig. 1 A) (Seals et al., 2000; Wurmser et al., 2000; van der Beek et al., 2019; Shvarev et al., 2022). Recently, mitochondrial relocalization was introduced as a powerful method to investigate the regulators and downstream interactors, as well as the docking and recruiting function of tethering proteins (Shin et al., 2017; Wong and Munro, 2014; Sanza et al., 2019; Schoppe et al., 2020; Gillingham et al., 2019). Here, we took a similar approach to study the role of VPS41 in mammalian cells.

**Figure S1.**
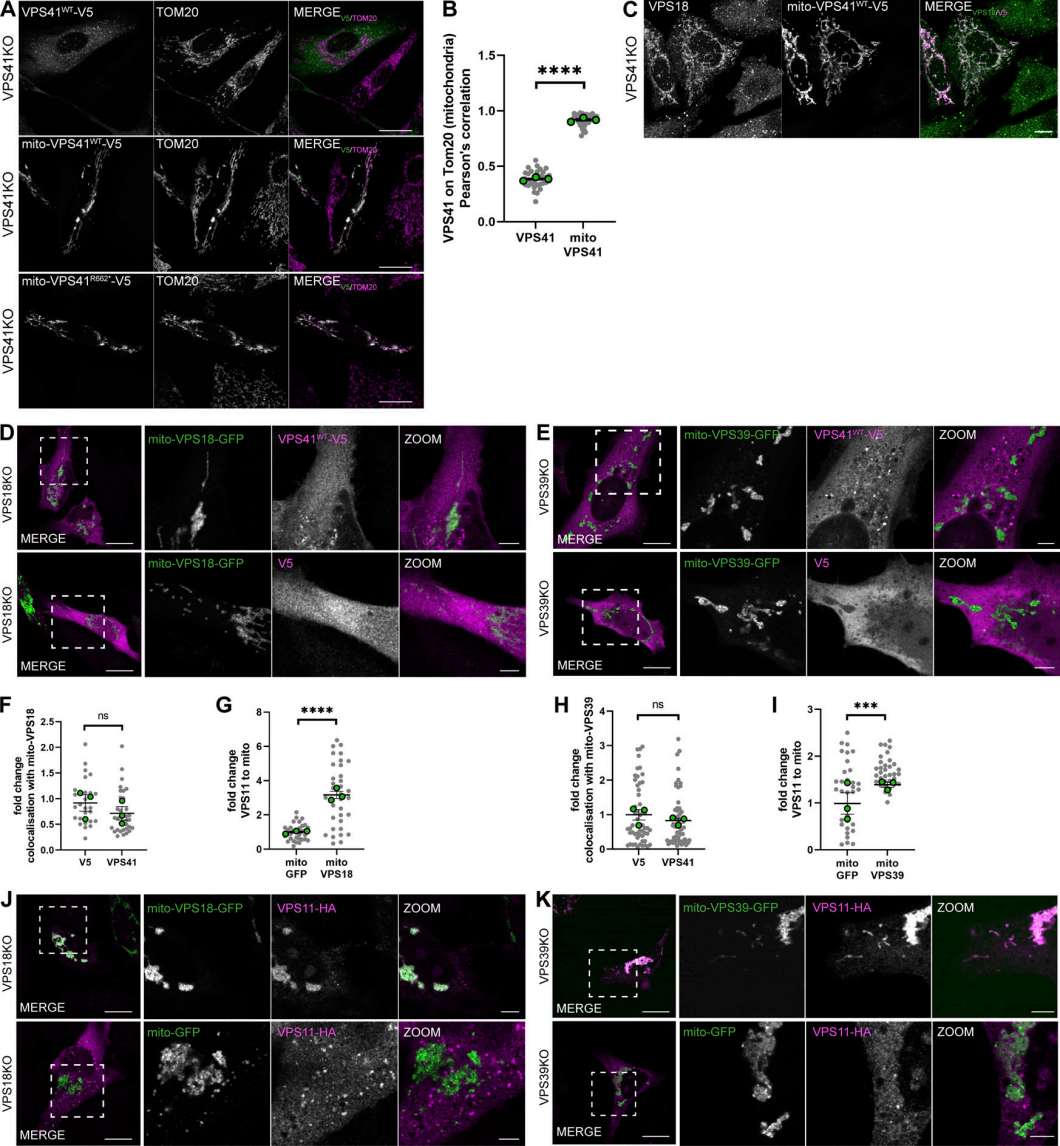
**Mito-VPS18 and mito-VPS39 are not able to recruit VPS41. (A)** Confocal images of HeLa^VPS41KO^ cells transfected with VPS41^WT^-V5, mito-VPS41^WT^-V5, or the truncation mutant mito-VPS41^R662*^-V5 (which lacks the RING domain) and labeled for V5 and endogenous TOM20. Both mito-VPS41 constructs were overlapping with TOM20-positive mitochondria. Scale bars: 20 μm. **(B)** Pearson’s correlation coefficient of anti-V5 (VPS41^WT^-V5 and mito-VPS41^WT^-V5) versus mitochondrial Tom20 as shown in A. Data show the mean ± SEM individual cell experimental replicate means, respectively. *n* ≥33. Statistical analysis was obtained using an unpaired *t* test, ****P < 0.0001. **(C)** HeLa^VPS41KO^ cells transfected with mito-VPS41^WT^-V5 and immunolabeled for endogenous VPS18 and V5 as described in the figure. Scale bars: 10 µm. **(D)** Confocal images of HeLa^VPS18KO^ cells co-expressing mito-VPS18-GFP and VPS41^WT^-V5 or empty vector V5 and immunostained for a V5 tag. Scale bars: 20 μm and 5 μm (inset). **(E)** HeLa^VPS39KO^ cells co-expressing mito-VPS39-GFP and VPS41^WT^-V5 or empty vector V5 and immunostained for a V5 tag, showing that mito-VPS39-GFP is not able to recruit VPS41^WT^-V5. Scale bars: 20 and 5 μm (inset) **(F)** Quantification of VPS41 ^WT^-V5 and empty vector V5 recruitment to mito-VPS18-GFP as shown in D. Mito-VPS18-GFP is not able to recruit VPS41. Quantification is expressed in fold change with value 1 to the negative control V5. Statistical significance was calculated using an unpaired *t* test, ns: not significant. *n* > 28. Data show the mean ± SEM and experimental replicate means, respectively. **(G)** Scatter plot quantification of VPS11-HA recruitment to mito-VPS18-GFP or negative control mito-GFP as shown in J. Mito-VPS18-GFP recruits VPS11. Quantification is expressed in fold change with value 1 to VPS11-HA signal on the mask of the negative control mito-GFP. Statistical significance was calculated using an unpaired *t* test, ****P < 0.0001. *n* > 30. Data show the mean ± SEM. **(H)** Quantification of VPS41 ^WT^-V5 and empty vector V5 recruitment to mito-VPS39-GFP as shown in E. Mito-VPS39-GFP is not able to recruit VPS41^WT^-V5. Quantification is expressed in fold change with value 1 given to the mitochondrial localization of the negative control empty vector V5. Statistical significance was calculated using an unpaired *t* test, ns: not significant. *n* > 30. Data show the mean ± SEM. **(I)** Scatter plot quantification of VPS11-HA recruitment to mito-VPS39-GFP or negative control mito-GFP as shown in K. Mito-VPS39-GFP is able to recruit VPS11. Quantification is expressed in fold change with value 1 to VPS11-HA signal on the mask of the negative control mito-GFP. Statistical significance was calculated using an unpaired *t* test, ***P < 0.001. *n* > 30. Data show the mean ± SEM. **(J)** Confocal images of HeLa^VPS18KO^ expressing VPS11-HA and either mito-VPS18-GFP or negative control mito-GFP as indicated in the figure. Cells were immunolabeled for an HA tag. Scale bars: 20 and 5 μm (inset). **(K)** Confocal images of HeLa^VPS39KO^ expressing VPS11-HA and either mito-VPS39-GFP or negative control mito-GFP. Immunostaining for an HA tag was performed. Scale bars: 20 and 5 μm (inset).

The N terminus of VPS41 contains a WD40, tetratricopeptide repeat (TPR)–like, and clathrin heavy-chain repeat (CHCR) domain. The C terminus contains a RING-H2 zinc finger domain, which is important for binding to the core subunits VPS18 and VPS33A (Harrington et al., 2012; Radisky et al., 1997; McVey Ward et al., 2001; Hunter et al., 2017). To relocalize VPS41 to mitochondria, we added the mitochondrial outer membrane protein translocase of the outer membrane 70 (TOM70) to a VPS41-APEX2-V5 construct previously made in our laboratory (van der Welle et al., 2021). The V5 tag was used for localization of the construct by immunofluorescence microscopy using anti-V5. We will hereafter refer to this construct as mito-VPS41^WT^-V5 or mito-VPS41 (Fig. 1 B). To test whether this tag resulted in mitochondrial relocalization, HeLa^VPS41KO^ cells were transiently transfected with mito-VPS41^WT^-V5 and prepared for confocal microscopy. Colocalization with Tom20 showed a complete overlap, indicating a full relocalization of mito-VPS41^WT^-V5 toward the mitochondrial surface (Fig. S1, A and B).

To control for the functionality of the construct, we tested whether mito-VPS41^WT^-V5 could recruit other subunits of the HOPS complex. The expression of VPS18-GFP in HeLa^VPS41KO^ cells expressing mito-VPS41^WT^-V5 showed that the two signals indeed largely overlapped on mitochondria (Fig. 1 C; for details on quantification, see the Materials and methods section). In addition, we observed a partial mitochondrial redistribution of endogenous VPS18 (Fig. S1 C). Of note, all cytosolic proteins exhibited a basal level of overlap with mitochondria in all conditions; therefore, the data are normalized for the control mito-V5 (Fig. 1 D). The experimental data represent the number of times the signal exceeded this background.

The N terminus of VPS41 contains a WD40, TPR-like, and CHCR domain. The C terminus contains a RING-H2 zinc finger domain, which is important for binding to the core subunits VPS18 and VPS33A (Harrington et al., 2012; Radisky et al., 1997; McVey Ward et al., 2001; Hunter et al., 2017). We recently showed that the patient-associated R662 mutation (VPS41^R662*^) encodes for a premature stop codon resulting in deletion of the RING domain (van der Welle et al., 2021). Despite its inability to assemble into the HOPS complex, this mutant still localizes to endo/lysosomes (van der Welle et al., 2021). To strengthen the demonstration of the functionality of mito-VPS41^WT^-V5 and test the role of the RING domain in the recruitment properties of VPS41, we added a TOM70 tag to a previously made VPS41^R662*^-APEX2-V5 construct (van der Welle et al., 2021) (hereafter called mito-VPS41^R662*^-V5) (Fig. 1 B) and transiently expressed this in HeLa^VPS41KO^ cells. Mito-VPS41^R662*^-V5 relocalized toward mitochondria to the same extent as mito-VPS41^WT^-V5 (Fig. S1 A). However, unlike mito-VPS41^WT^-V5, mito-VPS41^R662*^-V5 failed to recruit VPS18-GFP to mitochondria (Fig. 1, C and D).

To investigate vice versa whether other subunits of the HOPS complex can recruit VPS41, we fused TOM70 to the N terminus of VPS18-GFP (mito-VPS18-GFP) and VPS39-GFP (mito-VPS39-GFP) and co-expressed these constructs with VPS41^WT^-V5 or V5 in HeLa^VPS18KO^ and HeLa^VPS39KO^ cells (Fig. S1, D and E). Interestingly, neither mito-VPS18-GFP nor mito-VPS39-GFP recruited VPS41^WT^-V5 (Fig. S1, D–F and H) although they were both able to recruit the HOPS core subunit VPS11 (Fig. S1, G and I–K). These data indicated that VPS41 could recruit VPS18, but vice versa that VPS18 and VPS39 could not recruit VPS41. The inability of VPS18 to recruit VPS41 supports a previous suggestion by Khatter et al. (2015a) that HOPS assembly on lysosomal membranes depends on the presence of VPS41.

These results showed that mito-VPS41^WT^-V5 was able to recruit its natural interacting partner VPS18 and confirmed that the RING domain of VPS41 is essential for VPS18 recruitment and HOPS complex formation (Hunter et al., 2017), demonstrating the specificity of this recruitment assay.

### Mito-VPS41 recruits LAMP1-positive membranes

**Figure 2.**
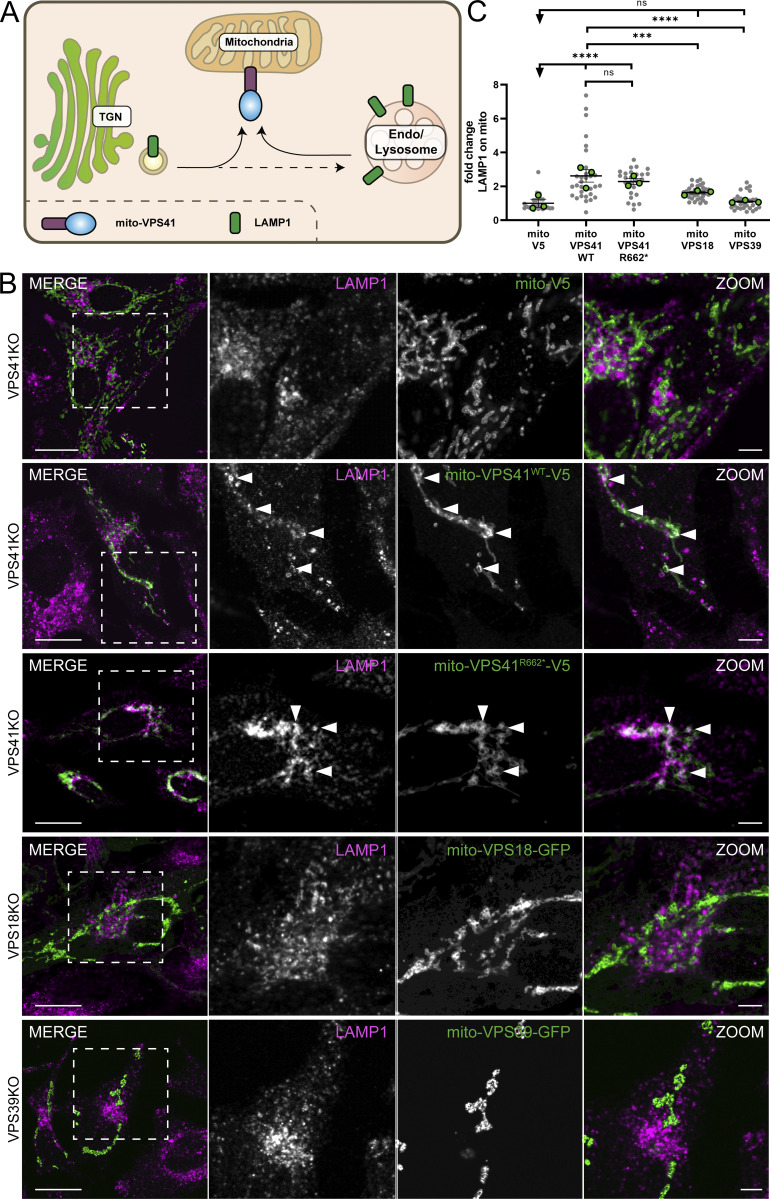
**Mito-VPS41 induces redistribution of LAMP1 to mitochondria. (A)** Schematic representation of the experimental setup. LAMP1-positive membranes recruited by mito-VPS41 could represent LAMP carriers and/or late endosomes/lysosomes. **(B)** Confocal immunofluorescence distribution of endogenous LAMP1 and negative control mito-V5, mito-VPS41^WT^-V5, or mito-VPS41^R662*^-V5 (stained with anti-V5) expressed in HeLa^VPS41KO^ cells. The bottom two rows show confocal immunofluorescence images of endogenous LAMP1 and mito-VPS18-GFP or mito-VPS39-GFP in HeLa^VPS18KO^ and HeLa^VPS39KO^ cells, respectively. Dashed boxes are enlarged in the next images. **(C)** Scatter plot quantification of LAMP1 recruitment to mitochondria upon the expression of mito-V5, mito-VPS41^WT^-V5, mito-VPS41^R662*^-V5, mito-VPS18-GFP, or mito-VPS39-GFP as shown in B. Scatter plot data are presented as the mean ± SEMreen dots for experimental replicate means. ****P < 0.0001, ***P < 0.001, ns: not significant. *n* ≥ 25. Statistical significance was obtained using Tukey’s multiple comparisons test. Scale bars: 20 or 5 µm (inset magnification).

To address the role of mammalian VPS41 in LAMP transport, we determined whether mito-VPS41^WT^-V5 was able to attract LAMP-containing membranes (Fig. 2 A). We first examined the fluorescence distribution of endogenous LAMP1 in HeLa^VPS41KO^ cells expressing mito-VPS41^WT^-V5 or the negative control mito-V5 (Fig. 2 B). Indeed, mito-VPS41^WT^ was able to redistribute LAMP1 to mitochondria, while the expression of the negative control mito-V5 had no effect (Fig. 2, B and C). Interestingly, the expression of the RING truncation mutant mito-VPS41^R662*^-V5 also resulted in relocation of LAMP1 to mitochondria (Fig. 2, B and C), indicating that the VPS41 RING domain or association between VPS41 and the HOPS complex was not required for LAMP recruitment.

To address the question whether recruitment of LAMP1-positive membranes was exclusive to VPS41 or a feature shared with other HOPS subunits, we then expressed the HOPS core subunit mito-VPS18-GFP or the other HOPS-specific subunit mito-VPS39-GFP in HeLa^VPS18KO^ and HeLa^VPS39KO^ cells, respectively (Fig. 2 B). Quantitative immunofluorescence analyses of endogenous LAMP1 showed that neither mito-VPS18-GFP nor mito-VPS39-GFP was able to reroute LAMP1 to mitochondria (Fig. 2, B and C).

Together, these data showed that mito-VPS41^WT^-V5 recruited LAMP1-positive membranes to mitochondria, independent of HOPS complex assembly. Moreover, recruitment of LAMP1 was specific for VPS41, because the expression of mito-VPS18-GFP or mito-VPS39-GFP had no effect on LAMP1 distribution. Since LAMP1 is a transmembrane protein, these findings implied that a subset of LAMP1-positive membrane organelles and/or vesicles are redirected to mitochondria upon mito-VPS41^WT^-V5 or mito-VPS41^R662*^-V5 expression.

### Mito-VPS41 recruits the small GTPase Arl8b

**Figure 3.**
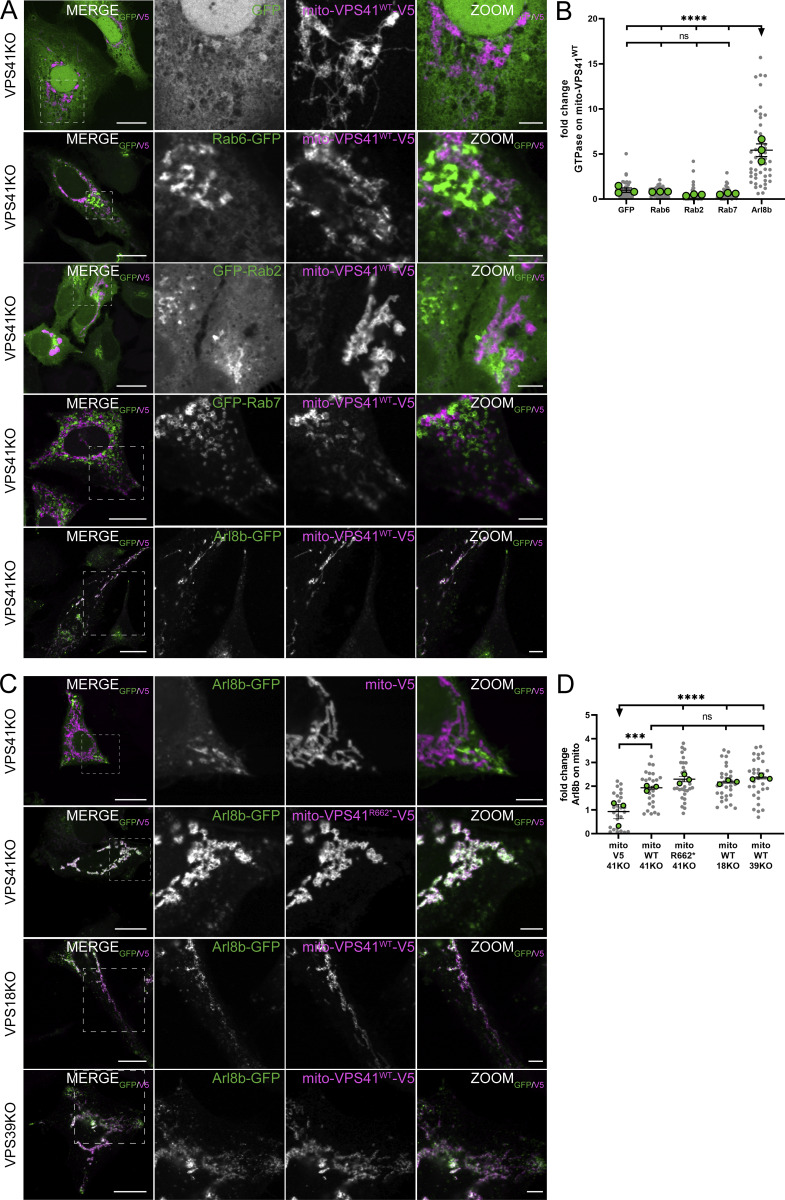
**Mito-VPS41 recruits the GTPase Arl8b. (A)** Confocal images of HeLa^VPS41KO^ cells cotransfected with mito-VPS41^WT^-V5 and GFP-Rab7, Arl8b-GFP, Rab2-GFP, Rab6-GFP, or GFP empty vector and labeled for V5. Dashed boxes are enlarged in the next images. **(B)** Quantification of mitochondrial recruitment of indicated GTPases upon the expression of mito-VPS41^WT^-V5. Values are the mean ± SEM. *n* > 40. Statistical significance was determined using Tukey’s multiple comparisons test. ****P < 0.0001, ns: not significant. **(C)** Confocal images of HeLa^VPS41KO^, HeLa^VPS18KO^, and HeLa^VPS39KO^ cells co-expressing Arl8b-GFP and mito-V5, mito-VPS41^R662*^-V5, or mito-VPS41^WT^-V5 and labeled for V5. **(D)** Quantification of mitochondrial recruitment of Arl8b-GFP by indicated constructs in C. Statistical significance was determined using Tukey’s multiple comparisons test. ****P < 0.0001, ***P < 0.001, ns: not significant, mean ± SEM. *n* > 25. Scale bars: 20 and 5 µm (inset magnification).

The membrane recognition process performed by tethering proteins requires interactions with small regulatory GTPases and their effectors (Spang, 2016). Rab2, Rab7 (Ras-related proteins Rab-2a and Rab-7a), and Arl8b (ADP ribosylation factor–like GTPase 8B), a member of the Arf-like (Arl) family proteins (Garg et al., 2011), have been indicated as direct or indirect binding partners of VPS41 (Ostrowicz et al., 2010; Ding et al., 2019; Khatter et al., 2015a). To identify the GTPase(s) involved in the delivery of LAMP membranes to mito-VPS41-V5, we assessed the recruitment of these GTPases using a similar assay as above. We expressed GFP-tagged constructs in HeLa^VPS41KO^ cells and investigated their putative recruitment by mito-VPS41^WT^-V5. We included a GFP empty vector and Rab6-GFP as negative controls since these do not interact with VPS41 (Fig. 3). Accordingly, these constructs remained disperse in the cytosol for GFP empty vector and predominantly in the Golgi area with no redistribution to mitochondria for Rab6-GFP.

**Figure S2.**
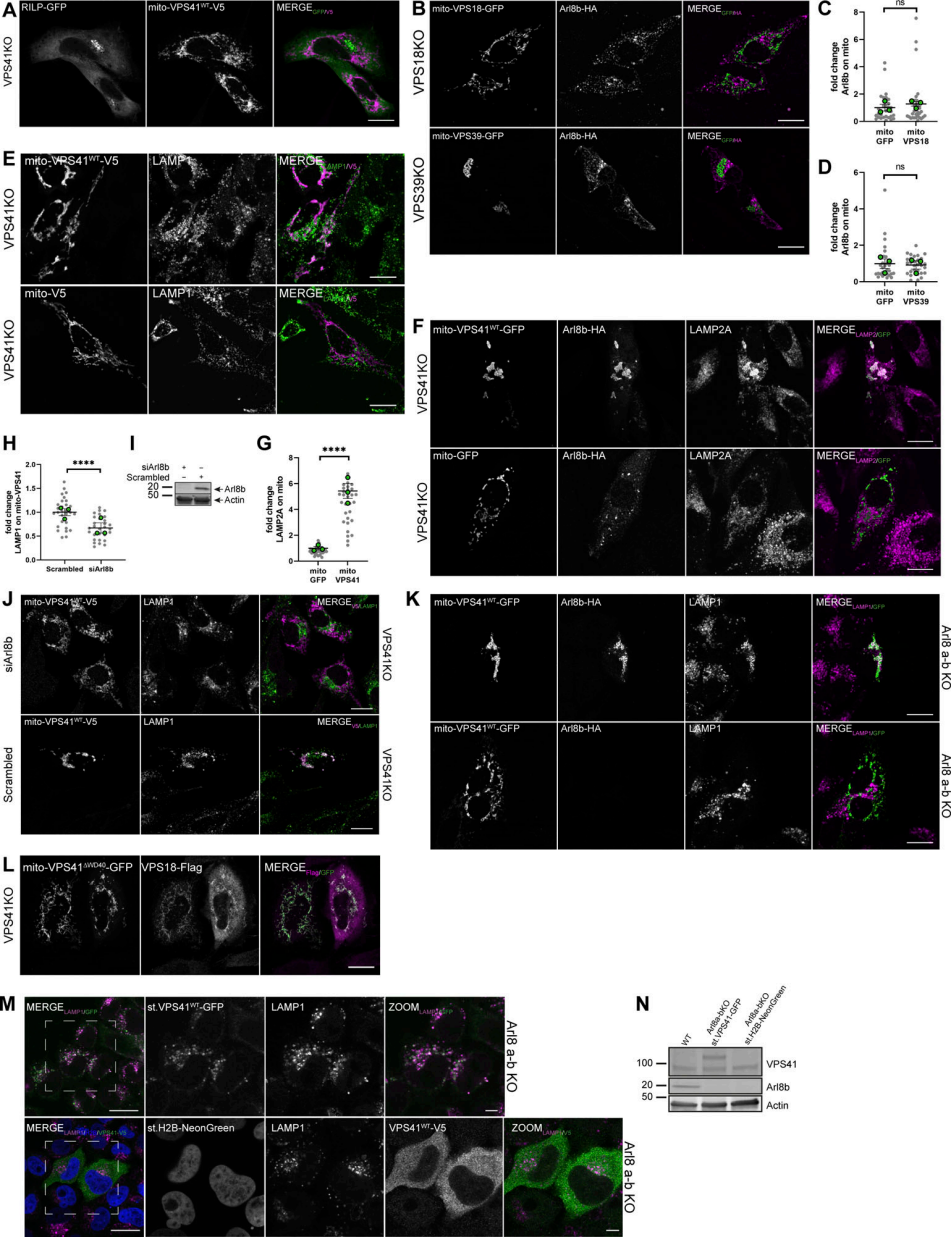
**Arl8b drives LAMP-positive membranes to mito-VPS41. (A)** Confocal image of HeLa^VPS41KO^ cells cotransfected with mito-VPS41^WT^-V5 and RILP-GFP. Cells were immunolabeled for a V5 tag. Scale bars: 20 µm. **(B)** Confocal microscopy of HeLa^VPS18KO^ and HeLa^VPS39KO^ cells cotransfected with Arl8b-HA and mito-VPS18-GFP or mito-VPS39-GFP as indicated in the figure. Immunostaining for an HA tag was performed. Scale bars: 20 µm. **(C and D)** Statistical quantification of Arl8b-HA recruitment on mitochondria harboring mito-VPS18-GFP or mito-VPS39-GFP as indicated. Statistical significance was obtained using an unpaired *t* test, ns: not significant. *n* > 25, mean ± SEM. **(E)** Confocal images of HeLa^VPS41KO^ cells single-transfected with mito-VPS41^WT^-V5 or mito-V5. Cells were immunolabeled for a V5 tag and endogenous LAMP1. Scale bars: 20 µm. **(F)** Confocal images of HeLa^VPS41KO^ cells cotransfected with Arl8b-HA and mito-VPS41^WT^-GFP or mito-GFP. Cells were immunolabeled for an HA tag and LAMP2A. Scale bars: 20 µm. **(G)** Statistical quantification of LAMP2A recruitment to mitochondria bearing mito-VPS41^WT^-GFP or mito-GFP. Unpaired *t* test comparison was used to determine statistical significance, ****P < 0.0001. *n* > 25, mean ± SEM. **(H)** Quantification of LAMP1 redistribution to mito-VPS41^WT^-V5 mitochondria in the presence (scrambled) or absence (siArl8b) of Arl8b. Data represent the mean ± SEM. Unpaired *t* test comparison was used to determine statistical significance, ****P < 0.0001. *n* > 25. **(I)** Immunoblot showing Arl8b expression in HeLa^VPS41KO^ treated with siArl8b or scrambled RNA. Actin was used as a loading control. **(J)** Fluorescence images of LAMP1 relocalization to mito-VPS41^WT^-V5 upon depletion of endogenous Arl8b (top) or non-targeting RNA (scrambled, bottom). Scale bar: 20 µm. **(K)** Confocal images of LAMP1 relocalization to mito-VPS41^WT^-GFP in HeLa^Arl8a-bKO^ cells rescued or not with Arl8b-HA. Scale bars: 20 µm. **(L)** Confocal image of HeLa^VPS41KO^ cells expressing mito-VPS41^∆WD40^-GFP and VPS18-Flag and immunostained for a Flag tag. Scale bars: 20 µm. **(M)** Confocal images of HeLa^Arl8a-bKO^ cells stably expressing either VPS41^WT^-GFP (top row) or H2B-NeonGreen (used as a negative control, bottom row). As a negative control, transiently overexpressed VPS41^WT^-V5 was used. Cells were labeled for endogenous LAMP1 and V5. Scale bar: 20 and 5 µm (inset). **(N)** Immunoblot showing the comparison of VPS41 and Arl8b protein levels between HeLa^WT^ versus HeLa^Arl8a-bKO^ cells stably expressing either VPS41^WT^-GFP or H2B-NeonGreen. Actin was used as a loading control. Source data are available for this figure: SourceData FS2.

Rab2 and Rab7 were also not recruited to mitochondria (Fig. 3), which was unexpected because in yeast, VPS41 interacts directly with the GTPase Ypt7p/Rab7 to regulate fusion events (Brett et al., 2008; Ostrowicz et al., 2010). Furthermore, in mammalian cells, it has been suggested that the VPS41–Rab7 interaction occurs by the Rab7 effectors RAB7-interacting lysosomal protein (RILP) (Lin et al., 2014; Van Der Kant et al., 2015) or Pleckstrin homology domain–containing protein family member 1 (PLEKHM1) (McEwan et al., 2015). We therefore also expressed RILP-GFP. This resulted in the characteristic accumulation of RILP-positive membranes in the perinuclear area (Cantalupo et al., 2001), which was not altered upon the expression of mito-VPS41^WT^-V5 (Fig. S2 A).

Conversely, we found that Arl8b-GFP was retargeted to mitochondria upon the expression of mito-VPS41^WT^-V5. Arl8b is known as an endo/lysosomal GTPase required for lysosomal fusion, positioning and trafficking via interaction with BLOC-one-related complex, kinesin-1 (Bagshaw et al., 2006; Hofmann and Munro, 2006; Pu et al., 2015; Khatter et al., 2015b), and SifA and kinesin-interacting protein (SKIP) (Rosa-Ferreira and Munro, 2011). To establish whether the recruitment of Arl8b by VPS41 (Fig. 3 B) was HOPS-dependent, we expressed the mito-VPS41^R662*^-V5, the construct that is unable to bind other HOPS subunits, in HeLa^VPS41KO^ cells. This resulted in a similar redistribution of Arl8b to mitochondria as seen after the expression of mito-VPS41^WT^-V5 (Fig. 3, C and D). Likewise, the co-expression of mito-VPS41^WT^-V5 and Arl8b-GFP in VPS18 or VPS39KO cells, i.e., in the absence of other HOPS components, induced relocalization of Arl8b to mitochondria (Fig. 3, C and D). Finally, we checked whether VPS18 or VPS39 could recruit Arl8b. This showed that neither mito-VPS18-GFP nor mito-VPS39-GFP induced relocalization of Arl8b to mitochondria (Fig. S2, B–D).

Collectively, our findings demonstrated that the small GTPase Arl8b was recruited by VPS41, whereas Rab2, Rab6, Rab7, and RILP were not. Strikingly, this recruitment property was not shared by VPS18 or VPS39 and did not require the presence of other HOPS subunits. This candidate approach may not have captured the entire spectrum of proteins recruited by VPS41. Future investigations employing unbiased proteomics techniques may uncover additional binding partners.

### Recruitment of LAMP-positive membranes by mito-VPS41 requires Arl8b

**Figure 4.**
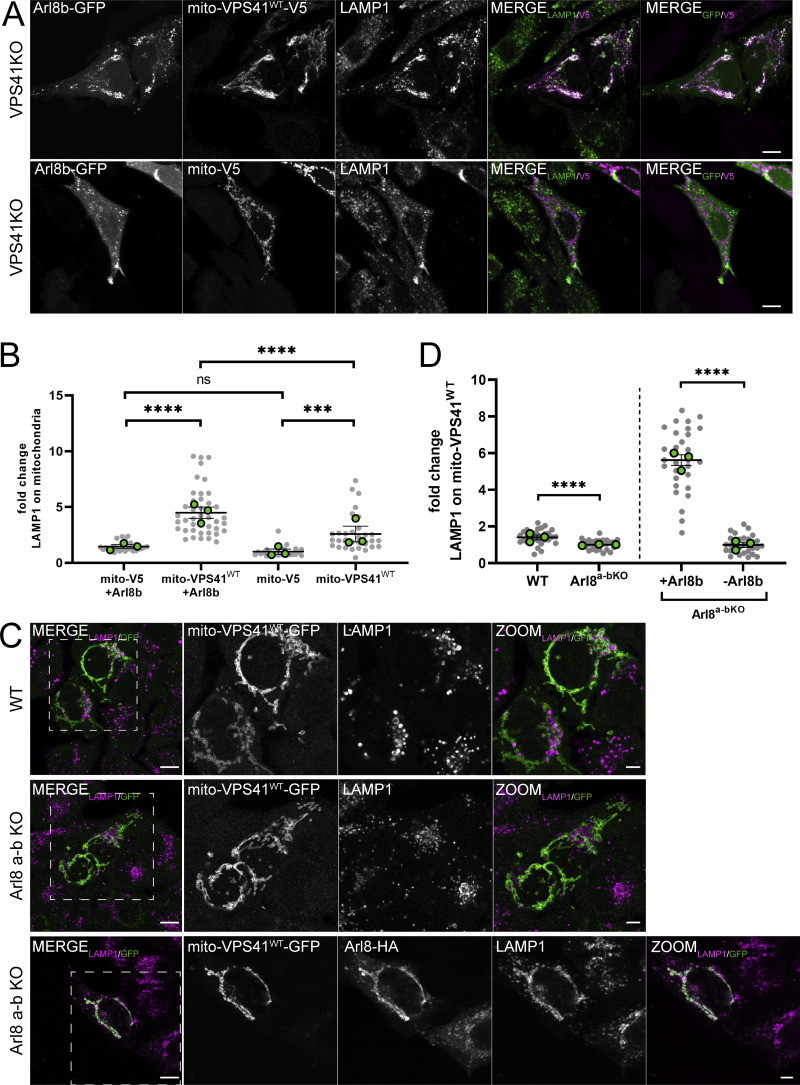
**Arl8b drives recruitment of LAMP1-positive membranes to mito-VPS41. (A)** Confocal images of HeLa^VPS41KO^ cells cotransfected with Arl8b-GFP and mito-VPS41^WT^-V5 or mito-V5 and immunolabeled for endogenous LAMP1 and V5. **(B)** Quantification of LAMP1 recruitment to mitochondria expressing mito-VPS41^WT^-V5 or mito-V5 in the presence or absence of Arl8b-GFP (see also Fig. S2, F–J). Scatter plot data represent the mean ± SEM. Statistical significance was determined using Tukey’s multiple comparisons test. ****P < 0.0001, ***P < 0.001, ns: not significant. *n* ≥ 25. Scale bars: 10 µm. **(C)** Confocal images of endogenous LAMP1 in HeLa^WT^ cells, HeLa^Arl8a-bKO^ cells, or HeLa^Arl8a-bKO^ cells rescued with Arl8b-HA (bottom) expressing mito-VPS41^WT^-GFP. **(D)** Quantification of mitochondrial redistribution of LAMP1 similar conditions as in C (see also Fig. S2 K). Data represent the mean ± SEM. Statistical significance was determined using an unpaired *t* test. ****P < 0.0001. *n* > 25. Scale bars: 10 and 5 µm (inset magnification).

To test whether Arl8b and LAMP1 were recruited together by mito-VPS41, we next co-expressed mito-VPS41^WT^-V5 and Arl8b-GFP in HeLa^VPS41KO^ cells. Using confocal microscopy and immunostaining of endogenous LAMP1, this resulted in a strong overlap between LAMP1 and Arl8b-GFP around the mitochondria (Fig. 4). Interestingly, we noticed that the overexpression of Arl8b significantly enhanced LAMP1 recruitment to mito-VPS41^WT^-V5 (Fig. 4, A and B; and Fig. S2 E). Similar results were obtained for LAMP2A (Fig. S2, F and G). In contrast, when Arl8b was overexpressed with mito-V5 (negative control), LAMP1-2 were not recruited to mitochondria (Fig. 4, A and B; and Fig. S2 G), confirming that the expression of mito-VPS41 was critically required to relocalize Arl8b/LAMP1 membranes to the ectopic mitochondrial location.

To investigate whether the interaction between Arl8b and VPS41 was instrumental in the trafficking of LAMP-positive membranes, we expressed mito-VPS41^WT^-GFP in the absence of Arl8a-b (Fig. 4 C). In HeLa^Arl8a-bKO^ cells (Fig. 4, C and D), mito-VPS41^WT^-V5 completely failed to recruit LAMP1 to mitochondria. Similar results were obtained upon silencing of Arl8b by siRNA (Fig. S2. H–J). Importantly, LAMP1 recruitment to mitochondria was restored upon reintroduction of Arl8b-HA in HeLa^Arl8a-bKO^ cells expressing mito-VPS41^WT^-GFP (Fig. 4, C and D; and Fig. S2 K). These results indicated a crucial role of Arl8b in the delivery of the LAMP1-positive membranes to mito-VPS41.

**Figure 5.**
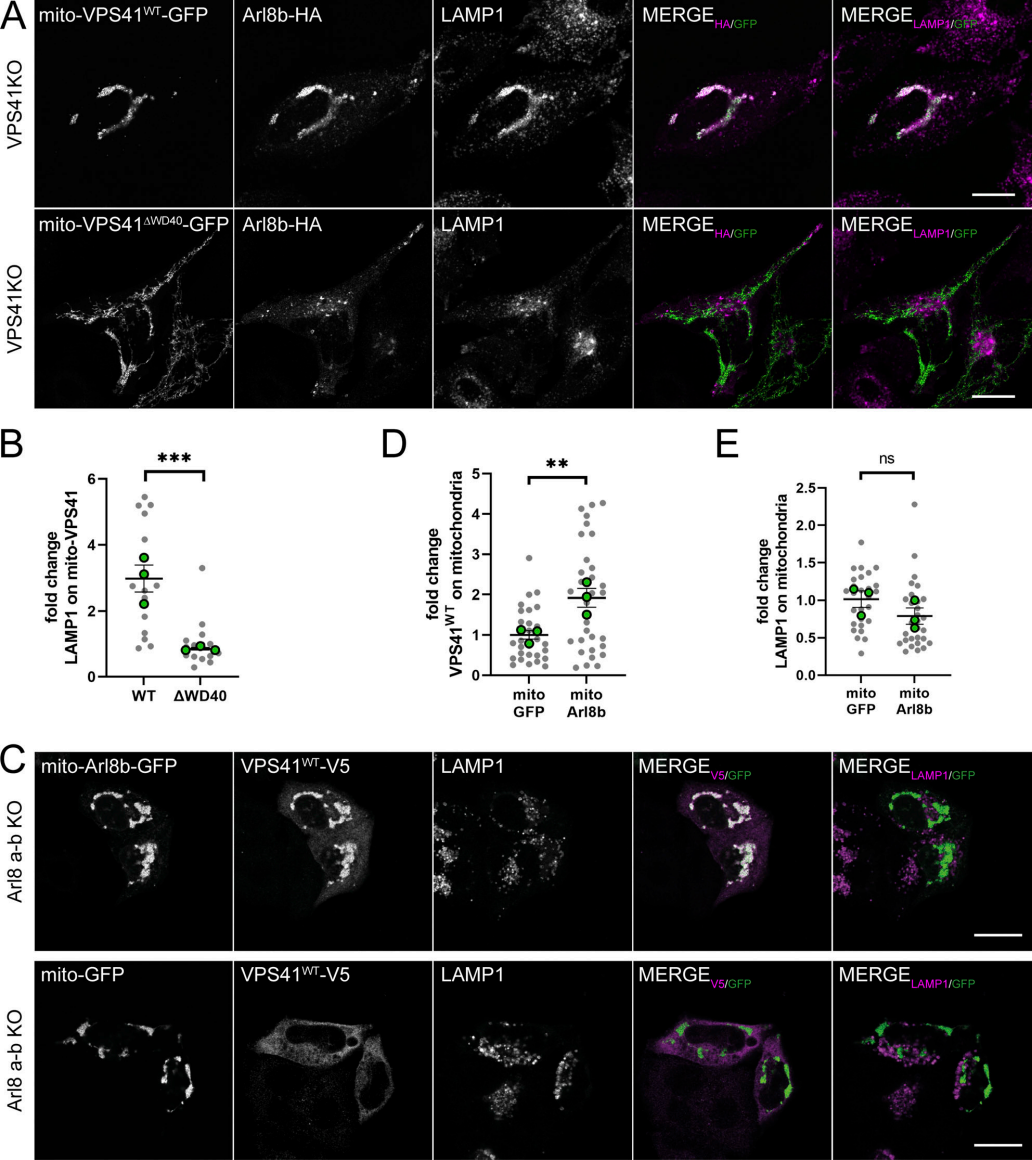
**Arl8b-binding WD40 domain of VPS41 is crucial for recruitment of LAMP1-positive membranes. (A)** Confocal images of HeLa^VPS41KO^ cells transfected with Arl8b-HA and mito-VPS41^∆WD40^-GFP (top), or mito-VPS41^WT^-GFP (bottom) and immunolabeled for endogenous LAMP1 and HA. **(B)** Quantification of LAMP1 recruitment to mitochondria upon mito-VPS41^∆WD40^-GFP or mito-VPS41^WT^-GFP expression. Statistical significance was obtained using an unpaired *t* test, ***P < 0.001. *n* = 15. Data represent the mean ± SEM. **(C)** Confocal images of VPS41^WT^-V5 recruitment to mito-Arl8b-GFP or mito-GFP in HeLa^Arl8a-bKO^ cells immunolabeled for V5 and endogenous LAMP1. **(D)** Quantification of VPS41^WT^-V5 overlapping mitochondria expressing mito-Arl8b-GFP or mito-GFP, respectively. **(E)** LAMP1 recruitment to mitochondria bearing mito-Arl8b-GFP or mito-GFP. Statistical analysis was obtained using an unpaired *t* test, **P < 0.01, ns: not significant. *n* > 25, mean ± SEM. Scale bars: 20 µm.

VPS41 is known to bind Arl8b via its WD40 domain (Khatter et al., 2015a). To test whether this domain was required for recruitment of LAMP1/Arl8b-positive membranes, we made a mito-VPS41 mutant lacking the WD40 domain, but with an intact RING domain (hereafter mito-VPS41^∆WD40^-GFP). The co-expression of mito-VPS41^∆WD40^-GFP and Arl8b-HA in HeLa^VPS41KO^ cells clearly showed that VPS41 without the WD40 domain failed to recruit LAMP1 and Arl8b to mitochondria (Fig. 5, A and B). In contrast, and as predicted by the presence of the RING domain, mito-VPS41^∆WD40^-GFP could still recruit VPS18 (Fig. S2 L).

Our data showed that recruitment of Arl8b to mitochondria was dependent on mito-VPS41 (Fig. 3). Vice versa, previous studies have shown that recruitment of VPS41 to endo/lysosomes is dependent on Arl8b (Garg et al., 2011; Marwaha et al., 2017; Khatter et al., 2015a). To test this further, we addressed whether Arl8b in a similar assay could recruit VPS41. We switched our experimental setup and fused Arl8b to TOM70 and GFP (mito-Arl8b-GFP). When expressed in HeLa^Arl8a-bKO^ cells together with VPS41^WT^-V5, mito-Arl8b-GFP redistributed a portion of VPS41^WT^-V5 to mitochondria (Fig. 5 C). Notably, and in line with previous reports (Garg et al., 2011; Marwaha et al., 2017; Khatter et al., 2015a), the remaining cytosolic pool of VPS41^WT^-V5 did not associate with LAMP1-positive endo/lysosomes that in this case lacked Arl8b. LAMP1 distribution remained unaltered upon the expression of mito-Arl8b-GFP. We next generated HeLa^Arl8a-bKO^ cells stably expressing low levels of VPS41-GFP. In contrast to cells with high VPS41 overexpression, in these cells VPS41 associated with LAMP1 puncta even in the absence of Arl8b (Fig. S2, M and N).

Together, these results showed that the presence of Arl8b on LAMP-positive membranes is required to enable recruitment and binding to mito-VPS41. The VPS41-WD40 domain is essential for this interaction, while the VPS41-RING domain is not involved. Our data confirmed previous studies (Khatter et al., 2015a) that Arl8b on endo/lysosomes was required for recruitment of VPS41; however, we found this was only the case when levels of VPS41 were high (overexpressed). Low (near endogenous) levels of VPS41 could associate with endo/lysosomes independent of Arl8b.

### Mito-VPS41 recruits small LAMP1- and Arl8b-positive vesicles

**Figure 6.**
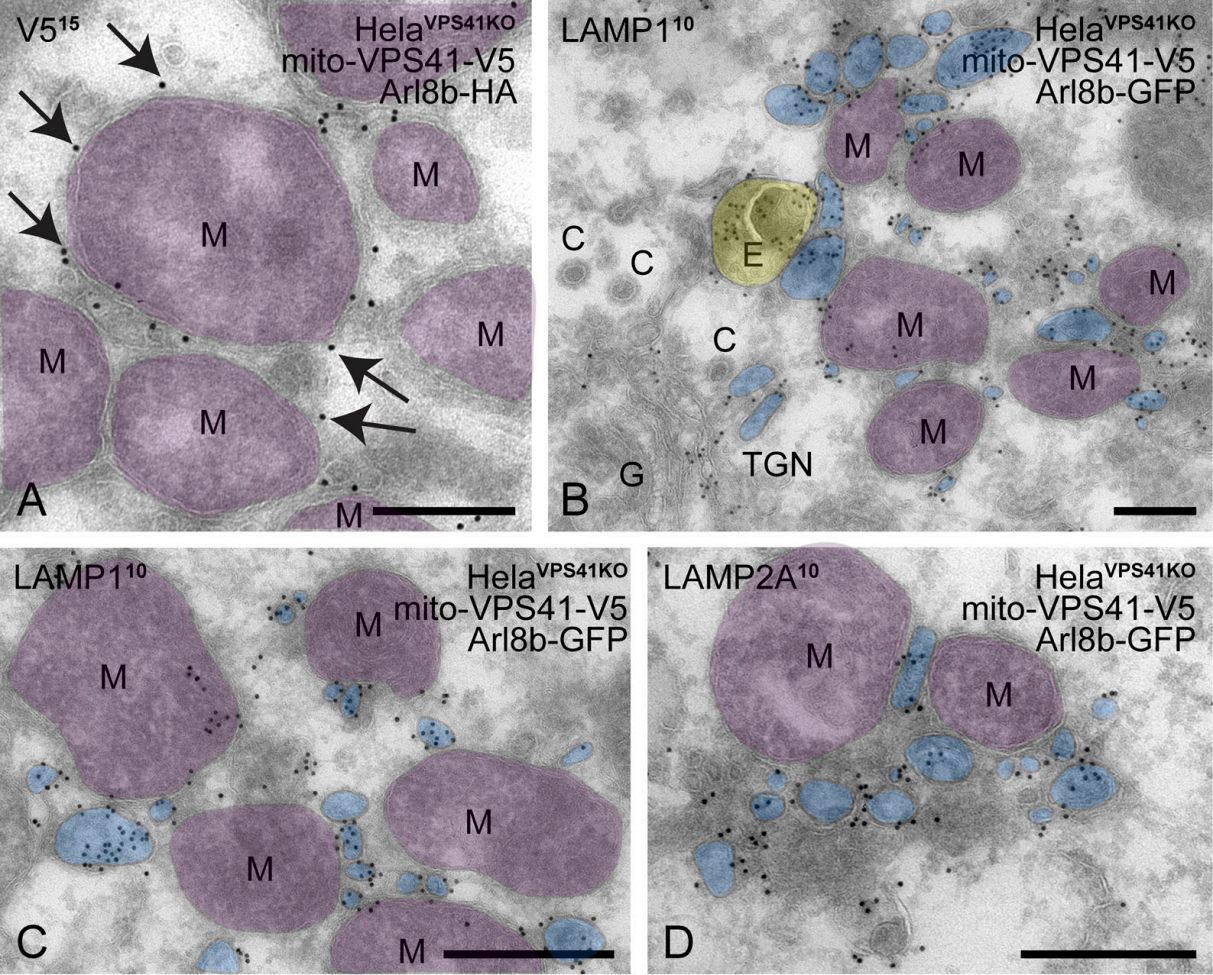
**Mito-VPS41–expressing cells recruit LAMP-positive, non-coated vesicles to mitochondrial electron micrographs with pseudocolored organelles. (A)** HeLa^VPS41KO^ cells transfected with mito-VPS41^WT^-V5 and Arl8b-HA, incubated with BSA^5^ (2 h) to mark endosomes and immunolabeled for V5 (15-nm gold). Arrows point to mito-VPS41^WT^-V5 present at the outer mitochondrial membranes (M, mitochondria, pseudocolored in purple). **(B and C)** HeLa^VPS41KO^ cells transfected with mito-VPS41^WT^-V5 and Arl8b-GFP and immunolabeled for LAMP1 (10-nm gold). **(B)** LAMP1 is seen in variably sized, non-coated vesicles (blue) in the TGN and around mitochondria (purple). Note the absence of LAMP1 from clathrin-coated (C) vesicles in the TGN area. **(C)** Higher magnification of LAMP1-positive, small vesicles (blue) surrounding mito-VPS41-V5–positive mitochondria. **(D)** Same cells as B and C but labeled for LAMP2A (10-nm gold), showing that the vesicles (blue) recruited by mito-VPS41 are also positive for LAMP2A. E, endosome (yellow); G, Golgi complex; M, mitochondrion (purple); vesicles (blue). Scale bars: 500 nm. For original EM images, see Fig. S3 A.

LAMP1 is a transmembrane protein mostly present in late endosomes and lysosomes, but a small pool is also present in early endosomes, plasma membranes, and TGN-derived LAMP carriers (Pols et al., 2013b). On the other hand, Arl8b has been described as an endo/lysosomal GTPase (Bagshaw et al., 2006; Hofmann and Munro, 2006; Schleinitz et al., 2023). To identify the nature of the LAMP1/Arl8b-positive membranes recruited by mito-VPS41, we co-expressed mito-VPS41^WT^-V5 and Arl8b-GFP in HeLa^VPS41KO^ cells and prepared these for immuno-EM. Using anti-V5 and gold labeling on ultrathin cryosections, we confirmed the mitochondrial localization of mito-VPS41^WT^-V5, showing a regular pattern of VPS41 representing gold particles at the outer mitochondrial membrane (Fig. 6 A). Notably, the mito-VPS41^WT^-V5–labeled mitochondria were often clustered together. This clustering has been previously observed in mito-targeting experiments of tethering factors and could either indicate a mitochondrial stress response and/or reflect tethering by mito-VPS41^WT^-V5 (Wong and Munro, 2014; Keren-Kaplan et al., 2022). We also found some late endosomes and lysosomes near mito-VPS41^WT^-V5–labeled mitochondria, but most membranes surrounding these mitochondria were small vesicles, with diameters ranging between 50 and 200 nm. Immunogold labeling of endogenous LAMPs showed that these vesicles were positive for both LAMP1 and LAMP2A (Fig. 6). Thus, the electron microscopy (EM) data indicated that mito-VPS41^WT^-V5 attracted a mixture of membranes, the majority being small-sized vesicles positive for LAMP1 and LAMP2A.

**Figure 7.**
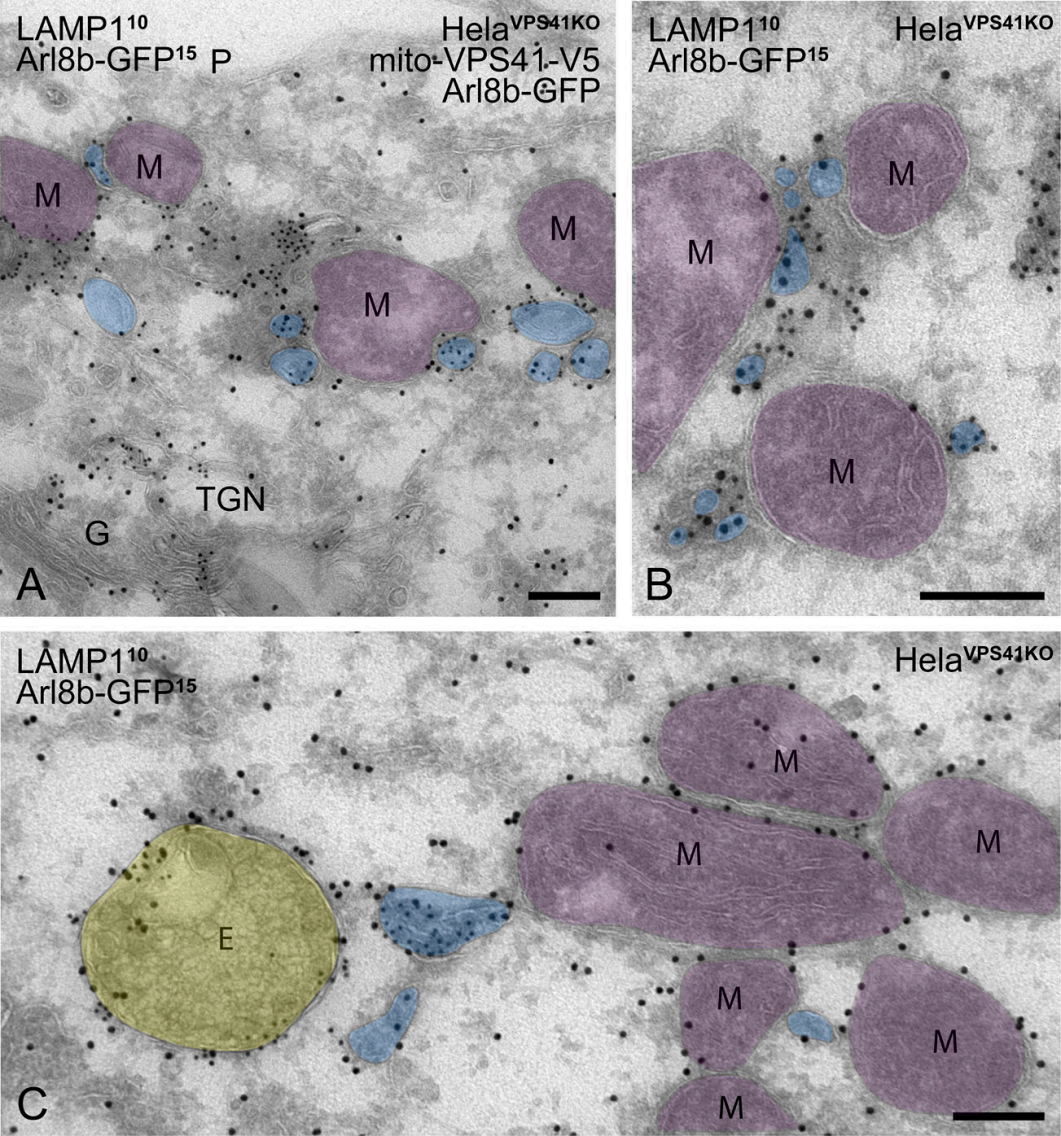
**LAMP1/Arl8b-positive vesicles accumulate around mitochondria in cells expressing mito-VPS41 HeLa** ^**VPS41KO**^ **cells transfected with mito-VPS41-V5 and Arl8b-GFP and immunolabeled for GFP and endogenous LAMP1. (A)** LAMP1 (10-nm gold) colocalizes with Arl8b (15-nm gold) on small vesicles (blue) in the TGN area and around mitochondria (M; purple). **(B)** Higher magnification of LAMP1/Arl8b-positive vesicles (blue) attached to mitochondria. **(C)** In addition to LAMP1-positive vesicles (blue), a cytosolic pool of Arl8b is present on mitochondria (M; purple). LAMP1 and Arl8b also colocalize on endosomes (E). E, endosome (yellow); G, Golgi complex; M, mitochondria (purple); vesicles (blue); TGN, trans-Golgi network; P, plasma membrane. Scale bars: 200 nm. For original EM images, see Fig. S3 B.

**Figure 8.**
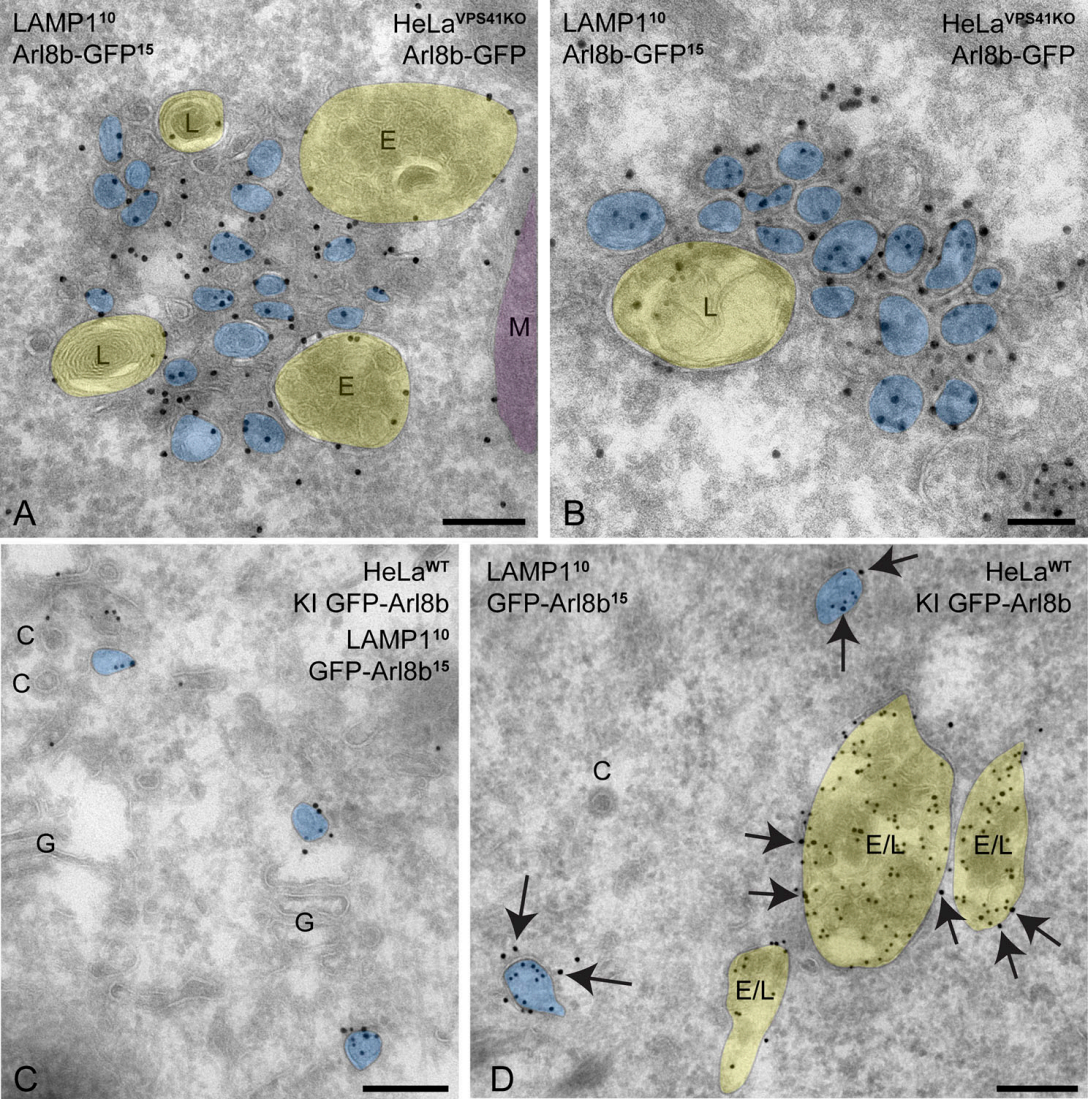
**LAMP1/Arl8b-positive vesicles in the absence of mito-VPS41. (A and B)** HeLa^VPS41KO^ cells transfected with Arl8b-GFP and immunolabeled for GFP and endogenous LAMP1. LAMP1 (10-nm gold) colocalizes with Arl8b (15-nm gold) on small vesicles (blue) surrounding endosomes and lysosomes (E and L, yellow). **(C and D)** HeLa^WT^ KI GFP-Arl8b, immunolabeled for GFP (15-nm gold) and endogenous LAMP1 (10-nm gold). Note the presence of Arl8b on endo/lysosomes (yellow) and on LAMP1-positive vesicles (blue) present in the Golgi region and in the vicinity of endo/lysosomes (E/L). Clathrin-coated vesicles (C) are devoid of label. C, clathrin-coated vesicle; Arrows in D point to Arl8b label (15-nm gold). E, endosome; G, Golgi; L, lysosome; M, mitochondrion. Scale bars: 200 nm. For original EM images, see Fig. S3 C.

To determine whether the LAMP-positive vesicles recruited by mito-VPS41^WT^-V5 were also positive for Arl8b, we performed double-immunogold labeling for Arl8b and LAMP1 in HeLa^VPS41KO^ cells expressing mito-VPS41^WT^-V5. This showed that the small-sized vesicles surrounding mito-VPS41^WT^-positive mitochondria contained both Arl8b and LAMP1 (Fig. 7). We then performed the same double labeling in the absence of mito-VPS41^WT^-V5. This resulted in disappearance of vesicles around mitochondria, but accumulation of similar small vesicles positive for Arl8b and LAMP1 in the proximity of late endosomes and lysosomes (Fig. 8).

Finally, to rule out that the vesicular localization of Arl8b was an overexpression artifact, we performed immuno-EM of GFP-tagged knock-in (KI) Arl8b HeLa cells (Fig. 8, C and D). This revealed endogenous Arl8b on Golgi and TGN membranes, the limiting membrane of late endosomes and lysosomes, and on non-coated vesicles often found in the vicinity of late endosomes/lysosomes. By morphology, these Arl8b vesicles were similar to LAMP carriers, and by double labeling, we confirmed that these vesicles were indeed positive for LAMP1. These data demonstrated the presence of endogenous Arl8b on LAMP carriers.

Together, these data showed that mito-VPS41 was able to especially reroute small, LAMP1-Arl8b vesicles to mitochondria, whereas in the absence of VPS41, these LAMP1-Arl8b vesicles accumulated around late endosomes and lysosomes. The presence of Arl8b on LAMP carriers was confirmed by double labeling of endogenous Arl8b and LAMP1.

### Arl8b/LAMP-positive vesicles recruited by mito-VPS41 are not of endocytic origin and do not contain the M6PR or AP3

**Figure 9.**
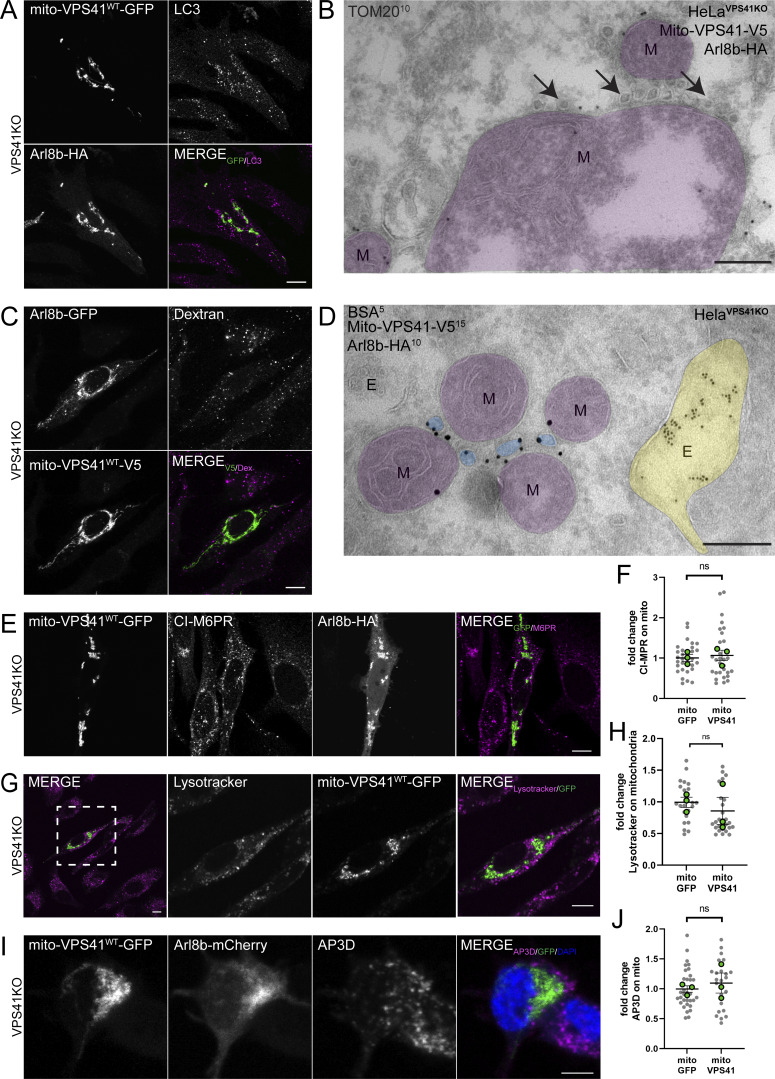
**Mito-VPS41 does not recruit LC3, CI-M6PR, AP3, or endo/lysosomal tracers. (A)** Confocal images of endogenous LC3. HeLa^VPS41KO^ cells expressing mito-VPS41^WT^-GFP and Arl8b-HA show no recruitment of LC3 to mitochondria. **(B)** Immuno-EM of HeLa^VPS41KO^ cells expressing mito-VPS41^WT^-V5 and Arl8b-HA and immunolabeled for endogenous Tom20 (10-nm gold). Vesicles accumulating around mitochondria (arrows) are negative for Tom20. For original EM images, see Fig. S3 D. **(C)** Confocal image of endocytosed dextran (2 h) in HeLa^VPS41KO^ expressing Arl8b-GFP and mito-VPS41^WT^-V5 and immunolabeled for V5 (10-nm gold). There is no recruitment of dextran to mito-VPS41^WT^-V5. **(D)** Immuno-EM of HeLa^VPS41KO^ cells expressing mito-VPS41^WT^-V5 and Arl8b-HA, incubated with BSA^5^, and labeled for HA and V5 (10- and 15-nm gold, respectively). Arl8b-HA–positive vesicles (blue) are devoid of endocytosed BSA^5^. E, endosome (yellow); M, mitochondria (purple). Scale bars: 500 nm. For original EM figures, see Fig. S3 D. **(E)** Confocal images showing CI-M6PR distribution in HeLa^VPS41KO^ cells expressing mito-VPS41^WT^-GFP and Arl8b-HA. **(F)** Quantification of CI-M6PR fluorescence on mitochondria shows similar distributions in cells expressing mito-VPS41^WT^-GFP or negative control mito-GFP (see also Fig. S3 E). Data represent means ± SEM. *n* > 25. Statistical significance was assessed using an unpaired *t* test, ns: not significant. **(G)** Confocal images of HeLa^VPS41KO^ cells expressing Arl8b-HA and mito-VPS41^WT^-GFP and incubated with LysoTracker (30 min) to visualize acidic compartments. **(H)** Quantification of overlap between LysoTracker and mitochondria shows no effect of mito-VPS41^WT^-GFP expression (see also Fig. S3 F). Data represent means ± SEM. *n* > 20. Significance was obtained using an unpaired *t* test, ns: not significant. **(I)** Confocal images showing endogenous localization of AP3D in PC12^VPS41KO^ cells transfected with Arl8b-mCherry and mito-VPS41^WT^-GFP. **(J)** Quantification of overlap between AP3D and mitochondria shows no effect of mito-VPS41^WT^-GFP (see also Fig. S3, G and H). Scatter plot represents the mean ± SEM. *n* ≥24. Statistical analysis was obtained using an unpaired *t* test, ns: not significant. Scale bars in I are 5 µm. Scale bars in A, C, E, and G are 10 µm and 5 µm (inset).

We subsequently performed a series of experiments to identify the nature of the Arl8b/LAMP1-positive vesicles recruited by mito-VPS41. To exclude that recruitment of mito-VPS41 induced mitochondrial vesiculation, we transfected HeLa^VPS41KO^ cells with mito-VPS41^WT^-GFP and Arl8b-HA and performed immunofluorescence of endogenous LC3 as a marker for macroautophagy (De Mazière et al., 2022). This clearly showed that LC3 was not present on the clustered mitochondria (Fig. 9 A). Moreover, by immuno-EM, we showed that Tom20, a marker for mitochondria and mitochondrial-derived vesicles, was absent from any of the mitochondrial-associated vesicles (Fig. 9 B) (Sugiura et al., 2014; Soubannier et al., 2012). These data implied that the mitochondrial-associated vesicles induced by mito-VPS41 expression were not caused by mitochondrial disintegration.

LAMP1 is found in different types of transport vesicles: endocytic vesicles derived from the plasma membrane (Janvier and Bonifacino, 2005), biosynthetic vesicles originating from the TGN (Pols et al., 2013b), and (AP3)-vesicles derived from early endosomes (Peden et al., 2004). To test whether the Arl8b/LAMP1-positive membranes recruited by mito-VPS41 contained endocytic cargo, we incubated HeLa^VPS41KO^ cells expressing Arl8b-GFP and mito-VPS41^WT^-V5 for 2 h with dextran–Alexa 568 (Fig. 9 C). Endocytosed dextran displayed a normal, dispersed pattern without any notable recruitment to mito-VPS41^WT^-V5/Arl8b-GFP mitochondria, indicating that most recruited membranes lacked endocytic cargo. Likewise, when we added BSA coupled to 5-nm gold particles (BSA^5^) to cells and prepared them for EM, we found 5-nm gold particles in endosomes but not in the mitochondrial-associated vesicles (Fig. 9 D). These data showed that the LAMP1/Arl8b-positive vesicles recruited by mito-VPS41 were not of endocytic origin.

**Figure S3.**
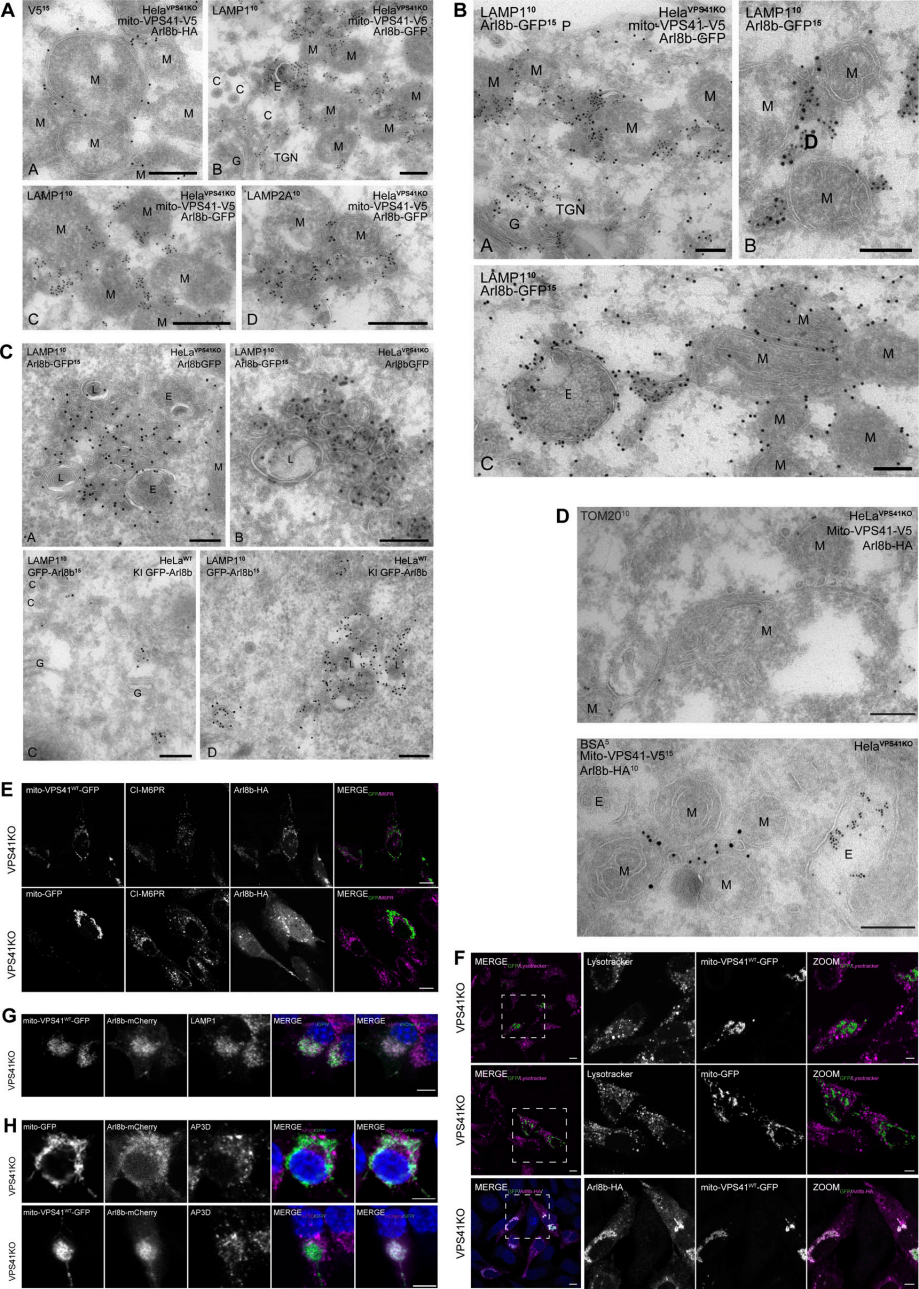
**Arl8b is on vesicles recruited by mito-VPS41. (A)** Original EM images of Fig. 6 without colored organelles. **(B)** Original EM images of Fig. 7 without colored organelles. **(C)** Original EM images of Fig. 8 without colored organelles. **(D)** Original EM images of Fig. 9 without colored organelles. **(E)** Confocal images showing the CI-M6PR distribution in HeLa^VPS41KO^ cells expressing Arl8b-HA together with either mito-VPS41^WT^-GFP or negative control mito-GFP. Scale bars: 10 µm. **(F)** Fluorescence image showing the LysoTracker dye distribution after 30 min of incubation in HeLa^VPS41KO^ cells expressing Arl8b-HA together with mito-VPS41^WT^-GFP (top) or mito-GFP (middle). The bottom line shows staining for Arl8b-HA in cells used for LysoTracker detection. Scale bars: 10 and 5 µm (inset). **(G)** Confocal images of PC12^VPS41KO^ cells expressing mito-VPS41^WT^-GFP and Arl8b-mCherry and immunolabeled for endogenous LAMP1. Scale bars: 5 µm. **(H)** Confocal images showing the endogenous localization of AP3D in PC12^VPS41KO^ cells transfected with Arl8b-mCherry and either mito-GFP or mito-VPS41^WT^-GFP. Scale bars: 5 µm.

We previously showed that LAMP carriers lack CI-M6PR, which mediates the transport of the majority of lysosomal enzymes from the TGN to endosomes (Höning et al., 1996; Doray et al., 2002; Pols et al., 2013b). To investigate whether CI-M6PR was recruited by mito-VPS41, we transiently cotransfected HeLa^VPS41KO^ cells with Arl8b-HA and mito-VPS41^WT^-GFP and assessed the localization of endogenous CI-M6PR by quantitative immunofluorescence. CI-M6PR distribution was not changed upon mito-VPS41^WT^-GFP expression (Fig. 9, E and F; and Fig. S3 E), indicating that recruited vesicles did not derive from the CI-M6PR/AP1 pathway. Moreover, since CI-M6PR is also present in endosomes, this was in agreement with our observation that only a small number of endosomes were recruited by mito-VPS41 (Fig. 6). Likewise, the distribution of LysoTracker-positive compartments was not markedly affected in cells expressing mito-VPS41^WT^-GFP (Fig. 9, G and H; and Fig. S3 F).

Previous studies in PC12 cells have suggested that VPS41 together with the adaptor protein 3 (AP3) is involved in the formation of secretory granules (Asensio et al., 2010, 2013). Furthermore, recent work in yeast showed that ectopic relocalization of VPS41 reroutes AP3-coated vesicles to mitochondria (Schoppe et al., 2020). Since AP3 levels in HeLa cells were beyond detection by immunofluorescence, we transfected PC12^VPS41KO^ cells with Arl8b-mCherry and either mito-VPS41^WT^-GFP or the negative control mito-GFP. Similar as in HeLa cells, mito-VPS41 expressed in PC12 cells redistributed LAMP1 and Arl8b to mitochondria (Fig. 9 I and Fig. S3 G). However, we found no change in AP3 distribution as compared to the negative control (Fig. 9, I and J; and Fig. S3 H). This observation showed that mammalian VPS41 was unable to recruit AP3 and that the LAMP1-positive membranes recruited by mito-VPS41 were devoid of AP3. These results reinforced the notion that the role of AP3 in trafficking is divergent from yeast to metazoans (Schoppe et al., 2020; Darsow et al., 2001; Rehling et al., 1999; Peden et al., 2004). Of note, due to their small and densely packed nature, PC12 cells exhibited a more diffuse fluorescence staining compared with HeLa cells.

Together, these data showed that the expression of mito-VPS41 did not induce mitochondrial vesiculation or mitophagy, did not lead to recruitment of endocytic, CI-M6PR or AP3-positive membranes, and did not lead to a significant redistribution of endo/lysosomal compartments to mitochondria.

### Arl8b/LAMP-positive vesicles recruited by mito-VPS41 are of biosynthetic origin

The morphology of LAMP1/Arl8b-positive vesicles recruited by mito-VPS41 matched our previous description of LAMP carriers as non-coated, irregularly formed, 50- to 200-nm-diameter vesicles (Pols et al., 2013b). To further confirm that they were indeed LAMP carriers, we used the Retention Using Selective Hooks (RUSH) system (Boncompain et al., 2012). We fused LAMP2A to a streptavidin-binding peptide (SBP) and added a mNeonGreen tag to visualize the subcellular localization of the construct (hereafter LAMP2A-RUSH). We co-expressed this with KDEL–streptavidin, which served as a hook to retain the construct in the ER. Upon the addition of biotin, the SBP–streptavidin bond was broken, resulting in a synchronous wave of newly synthesized LAMP2A exiting the ER and traveling to its final destinations.

**Figure 10.**
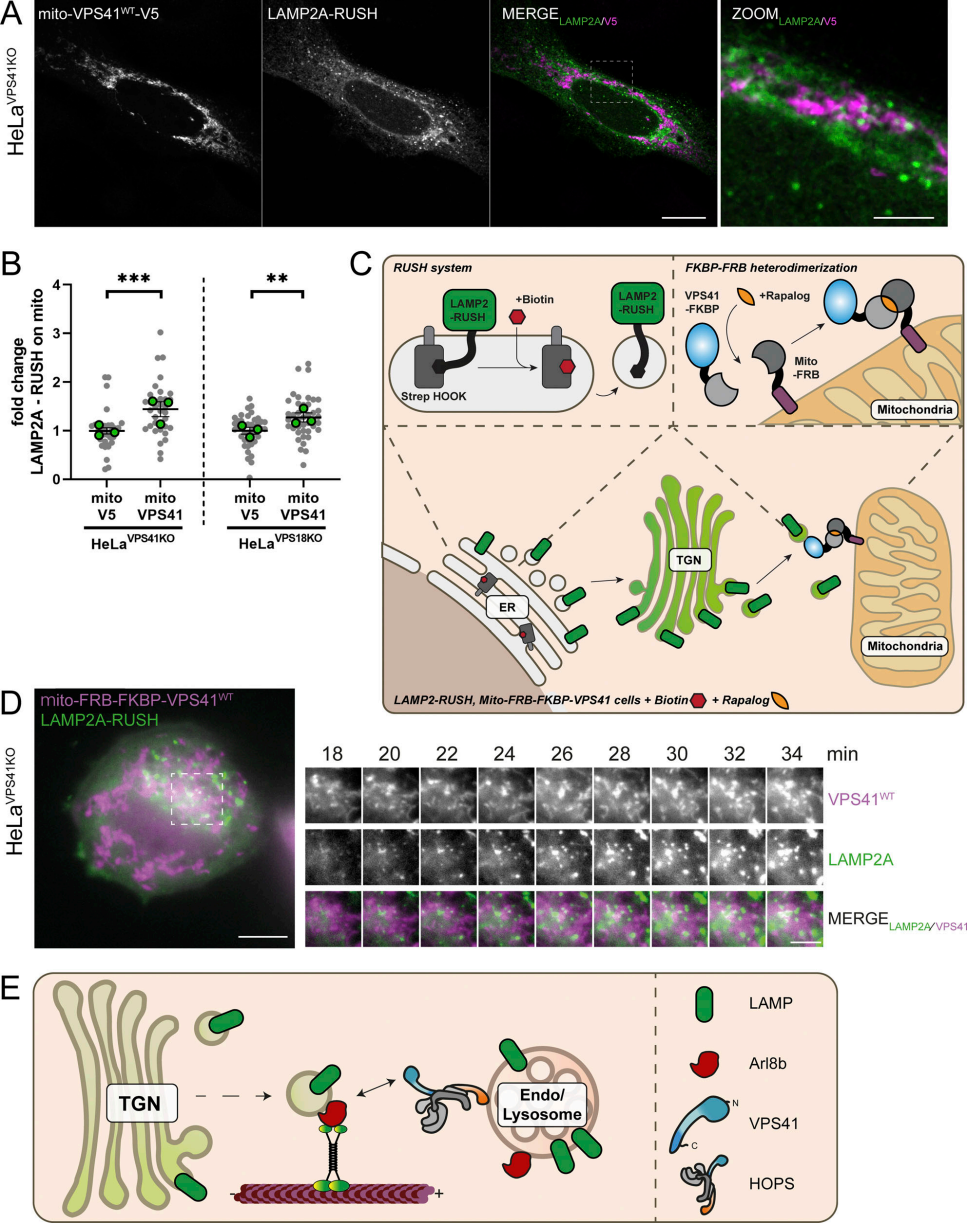
**Mito-VPS41 recruits LAMP2A-positive vesicles originating from the TGN. (A)** HeLa^VPS41KO^ cells expressing mito-VPS41^WT^-V5 and LAMP2A-RUSH 1.5 h after incubation with biotin. Scale bar: 10 and 5 µm (inset magnification). **(B)** Quantification of LAMP2A-RUSH recruitment to mitochondria expressing mito-VPS41^WT^-V5 versus negative control mito-V5, in HeLa^VPS41KO^ or HeLa^VPS18KO^ cells, respectively. Statistical analysis was obtained using an unpaired *t* test, **P < 0.01, ***P < 0.001. *n* > 30. Data show means ± SEM. **(C)** Schematic representation of synchronized LAMP2A release using the RUSH system and FRB-VPS41^WT^-Halo recruitment to mitochondria via the heterodimerization of the FKBP-FRB system. **(D)** HeLa^VPS41KO^ cells stably expressing mito-mCherry-FRB (magenta) transfected with FKBP-VPS41^WT^-Halo and LAMP2A-RUSH (green) 24 min after treatment with rapalog and biotin. The right panel shows representative time-lapse images of LAMP2A-RUSH–positive vesicles (green) that accumulate on mitochondria bearing mito-FRB-FKBP-VPS41^WT^ (magenta) (see also Figs. S4 and S5). **(E)** Summarizing model of LAMP carrier recruitment to late endosomes. After TGN release, LAMP carriers travel through Arl8-motor protein–mediated transport to late endosomes and are recruited by the interaction of Arl8b with the WD40 domain of VPS41. This enables local input and consequently increased concentrations of lysosomal membrane proteins. Subsequent fusion of late endosomes with lysosomes is enabled by the HOPS complex.

**Figure S4.**
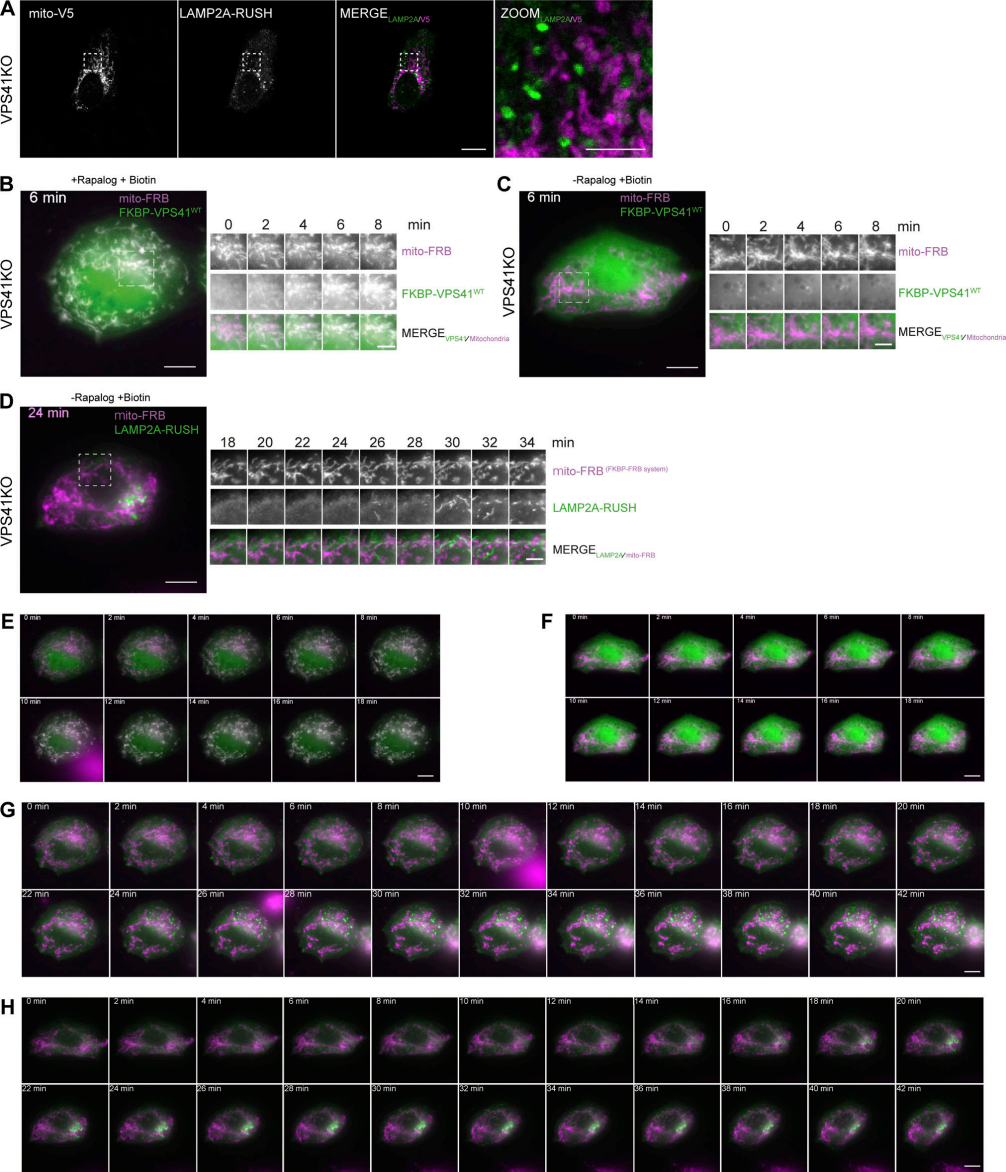
**LAMP2A-RUSH biosynthetic vesicles are recruited by mito-VPS41. (A)** Fluorescence image of HeLa^VPS41KO^ cells expressing mito-V5 and LAMP2A-RUSH after 1.5 h of incubation with biotin, used as a negative control for RUSH experiment in Fig. 10, A and B. Scale bar: 10 and 5 µm (inset). **(B)** Fluorescence image of HeLa^VPS41KO^ cells stably expressing mito-mCherry-FRB transfected with FKBP-VPS41^WT^-Halo and LAMP2A-RUSH. Image shows localization of mito-mCherry-FRB (magenta) and FRB-VPS41^WT^-FKBP (green) after 6 min of A/C heterodimerizer and biotin treatment (top). Representative time-lapse images of VPS41 recruitment to mitochondria (on the right). Scale bar: 10 and 5 µm (inset). **(C)** Fluorescence image at minute 6 of negative control where cells were only treated with biotin (top). Representative time-lapse images of VPS41 and mitochondria (on the right). Scale bar: 10 and 5 µm (inset). **(D)** Fluorescence image of negative control as above after 24 min of treatment with biotin (top). Representative time-lapse images of LAMP2A-RUSH–positive vesicles and mitochondria expressing mito-mCherry-FRB. No accumulation of LAMP2A on mitochondria was observed (bottom). Scale bar: 10 and 5 µm (inset). **(E–G)** Time-lapse panel of (E) FKBP-VPS41^WT^-Halo and (G) LAMP2A-RUSH recruitment to mito-mCherry-FRB upon treatment with rapalog and biotin. **(F–H)** Time-lapse panel of (F) FKBP-VPS41^WT^-Halo and (H) LAMP2A-RUSH and mito-mCherry-FRB upon treatment with only biotin. Scale bar: 10 µm.

HeLa^VPS41KO^ cells expressing the LAMP2A-RUSH construct were cotransfected with Arl8b-HA and either mito-VPS41^WT^-V5 or the negative control mito-V5. After the addition of biotin for 1.5 h, we found significant recruitment of LAMP2A-RUSH by mito-VPS41, but not by the control construct (Fig. 10, A and B; and Fig. S4 A). In line with Fig. 2, newly synthesized LAMP2A-RUSH was also recruited in HeLa^VPS18KO^ cells (Fig. 10 B), i.e., independent of HOPS complex assembly. Of note, while the recruitment of LAMP2A-RUSH vesicles by mito-VPS41 was significant compared with control conditions, the level of recruitment was less striking than observed for the total pools of endogenous LAMP1 or LAMP2A (see, e.g., Figs. 2, 3, and 4; and Fig. S2 K). A likely explanation for this discrepancy is the duration of the experiments: 1.5-h expression of LAMP2A-RUSH versus overnight recruitment of total LAMP levels. Moreover, in the RUSH experiments, mito-VPS41 was expressed several hours before LAMP2A-RUSH release, which allowed prior recruitment of endogenous LAMP carriers to mitochondria, possibly interfering with binding of LAMP2A-RUSH carriers.

**Figure S5.**
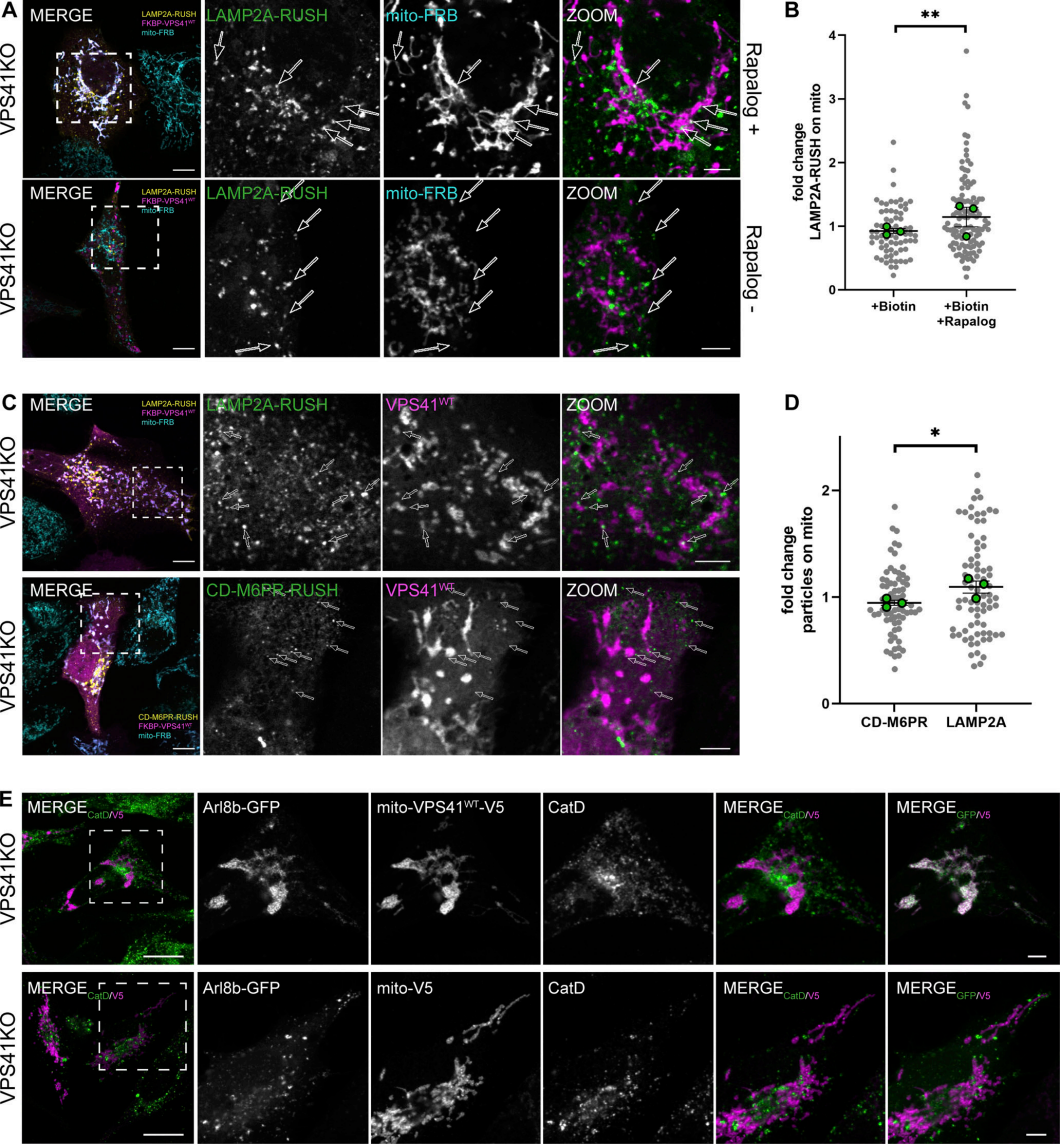
**Mito-VPS41 recruits biosynthetic LAMP2A-RUSH but not CD-M6PR-RUSH and CatD. (A)** Confocal images of HeLa^VPS41KO^ cells stably expressing mito-mCherry-FRB transfected with FKBP-VPS41^WT^-Halo and LAMP2A-RUSH after 45 min of treatment with biotin and rapalog (top) or only biotin (bottom) used as a negative control. Arrows indicate LAMP2-RUSH vesicles and their overlap with mito-mCherry-FRB images. Scale bar: 10 and 5 µm (inset magnification). **(B)** Scatter dot plot showing the quantification of the mitochondrion recruitment of LAMP2A-RUSH 45 min after biotin addition as shown in A. Quantification is expressed in fold change with value 1 given to LAMP2A-RUSH signal on the mask of the mitochondria expressing mito-mCherry-FRB not treated with rapalog used as a negative control. Statistical significance was calculated using an unpaired *t* test, **P < 0.01. *n* > 70, mean ± SEM. Grey dots and green dots represent individual cell data points and experimental replicate means, respectively. **(C)** Confocal images of HeLa^VPS41KO^ cells stably expressing mito-mCherry-FRB transfected with FKBP-VPS41^WT^-Halo and LAMP2A-RUSH (top) or CD-M6PR-RUSH (bottom) 45 min after treatment with biotin and rapalog. Arrows indicate LAMP2-RUSH or CD-M6PR-RUSH vesicles and their overlap with mito-mCherry-FRB images. Scale bar: 10 and 5 µm (inset). **(D)** Quantification of mitochondrion recruitment of LAMP2A-RUSH and CD-M6PR-RUSH 45 min after biotin treatment as shown in C. Quantification is expressed in fold change with value 1 to CD-M6PR-RUSH signal on the mask of the mitochondria expressing mito-mCherry-FRB treated with rapalog and biotin used as a negative control. Statistical significance was calculated using an unpaired *t* test, *P < 0.01. *n* > 70, mean ± SEM. **(E)** Confocal images showing the CatD distribution in HeLa^VPS41KO^ expressing Arl8b-GFP together with either mito-VPS41^WT^-V5 or negative control mito-V5. Scale bar: 20 µm.

To explore the dynamics of LAMP2A-RUSH association with mitochondria, we designed a method to target VPS41 to the mitochondria exclusively upon activation of the RUSH system and the release from the Golgi/TGN in live cells. We generated a HeLa^VPS41KO^ cell line stably expressing mito-mCherry-FRB, which was cotransfected with FKBP-VPS41^WT^-Halo and LAMP2A-RUSH. We then performed simultaneous redistribution of VPS41 to mitochondria via rapalog-induced heterodimerization of the FKBP-FRB subunits, and LAMP2A-RUSH release from the ER by adding biotin (Fig. 10 C). VPS41 was recruited to mitochondria <5 min after treatment with rapalog (Fig. S4 B). Although variable, upon adding biotin to the medium, we on average observed an accumulation of LAMP2-RUSH in the Golgi region between 15 and 20 min after release (Videos 1 and 2). Strikingly, already after 25 min, we observed accumulation of LAMP2A-RUSH on VPS41-positive mitochondria (Fig. 10 D; and Videos 3 and 4). As a control, we released LAMP2A-RUSH, but kept VPS41 in its original distribution by treating cells only with biotin (Fig. S4 C; and Videos 5 and 6). In these cells, we found no accumulation of LAMP2A-RUSH vesicles on mitochondria (Fig. S4 D; and Fig. S5, A and B).

**Video 1.** **Time-lapse imaging of Golgi accumulation and release of LAMP2A-RUSH upon biotin treatment.** HeLa^VPS41KO^ transfected with LAMP2A-RUSH were imaged live every 2 min after biotin treatment (Time 0). Movie playback 2 fps. Scale bar: 10 µm.

**Video 2.** **Time-lapse imaging of Golgi accumulation and release of LAMP2A-RUSH upon biotin treatment.** HeLa^VPS41KO^ transfected with LAMP2A-RUSH were imaged live every 2 min after biotin treatment (Time 0). Movie playback 2 fps. Scale bar: 10 µm.

**Video 3.** **Time-lapse imaging of LAMP2A-RUSH accumulation on mitochondria bearing FKBP-VPS41.** HeLa^VPS41KO^ cells stably expressing mito-FRB transfected with FKBP-VPS41^WT^-Halo and LAMP2A-RUSH were imaged every 2 min after treatment with biotin and rapalog. Movie playback 2 fps. Scale bar: 10 µm.

**Video 4.** **Time-lapse imaging of FKBP-VPS41** ^**WT**^ **recruitment to mitochondria bearing mito-FRB.** This video shows the recruitment of FKBP-VPS41^WT^-Halo to mito-FRB upon treatment with biotin and rapalog. Movie playback 1 fps. Scale bar: 10 µm.

**Video 5.** **LAMP2A-RUSH vesicles do not accumulate on mitochondria in negative control.** HeLa^VPS41KO^ cells stably expressing mito-FRB transfected with FKBP-VPS41^WT^-Halo and LAMP2A-RUSH were treated only with biotin and imaged every 2 min. Movie playback 2 fps. Scale bar: 10 µm.

**Video 6.** **Time-lapse imaging of FKBP-VPS41** ^**WT**^ **-Halo in negative control.** This video shows that FKBP-VPS41^WT^-Halo is not recruited to mitochondria bearing mito-FRB when treated with biotin alone without rapalog. Movie playback 1 fps. Scale bar: 10 µm.

Next, to control for the specificity of recruitment of LAMP2A-RUSH by mito-VPS41, we performed studies using a CD M6PR-RUSH construct (CD-M6PR-RUSH). Previously, we showed that the CI M6PR, which exits the TGN in similar vesicles as CD-M6PR (Klumperman et al., 1993), was absent from LAMP carriers. Concomitantly, CD-M6PR-RUSH was not recruited to mitochondria by mito-VPS41, reinforcing the distinct behavior of these vesicles in cellular transport and stressing the specificity of recruitment of LAMP carriers by VPS41 (Fig. S5, C and D).

Together, the RUSH experiments demonstrated that mito-VPS41 specifically recruited LAMP-positive vesicles originating from the TGN.

## Discussion

VPS41 is a well-characterized component of the HOPS complex, which is required for fusion events between lysosomes and late endosomes or autophagosomes, controlling the final and defining steps in cargo degradation (Radisky et al., 1997; Nickerson et al., 2009). In addition, VPS41 is required for the trafficking of biosynthetic vesicles, known as LAMP carriers, which transport lysosomal membrane proteins from the TGN to late endosomes (Pols et al., 2013b). However, the precise molecular mechanism by which VPS41 functions in this biosynthetic pathway and how this relates to HOPS complex function has remained elusive. In this study, we present evidence indicating that ectopically localized VPS41 serves as a destination target for LAMP carriers and that recruitment is dependent on the presence of the small GTPase Arl8b on the LAMP carriers, as well as an intact Arl8b-binding domain in VPS41. Importantly, the interaction between Arl8b and ectopically localized VPS41 did not require the presence of any of the other HOPS components and we found that recruitment of LAMP carriers was not shared by other HOPS subunits. When extrapolated to physiological conditions, these data imply a specific role of mammalian VPS41 within the HOPS complex, to recruit biosynthetic LAMP carriers to late endosomes and position them for subsequent HOPS-dependent fusion. Moreover, our data disclose an unexpected role of the endo/lysosomal GTPase Arl8b, which is required on the TGN-derived LAMP carriers to allow recruitment by VPS41. In Fig. 10 E, we propose a model illustrating the role of Arl8b and VPS41 in the transport of LAMP carriers in relation to the HOPS complex and endo/lysosome biogenesis.

The technique of ectopic relocalization, involving the artificial expression of proteins in locations where they do not naturally occur, is particularly valuable for dissecting in situ protein interactions, especially for protein complexes such as HOPS (Wong and Munro, 2014; Sanzà et al., 2019; Shin et al., 2020; Keren-Kaplan et al., 2022), since it enables to study interaction properties of individual subunits. Mito-VPS41^WT^ successfully relocalized toward the mitochondrial surface (Fig. S1 A) and was able to recruit its natural interacting partner VPS18 via its RING domain, demonstrating the specificity of the assay. Strikingly, we found that mito-VPS41 also recruited LAMP1-positive membranes. To determine whether the HOPS complex was required for this recruitment, we made use of a patient-derived VPS41^R662*^ truncation mutant that lacks the RING domain and cannot bind other subunits of the HOPS complex. In addition, we performed VPS41 recruitment assays in cells knocked out for the HOPS core component VPS18 or the other HOPS-specific subunit VPS39. This showed that recruitment of LAMP1-positive membranes by mito-VPS41 did not require the VPS41 RING domain or the presence of other HOPS components. Moreover, mito-VPS18 and mito-VPS39 were unable to recruit LAMP1 membranes, indicating that this recruitment function is a unique feature for VPS41.

Surprisingly, we found that recruitment of LAMP1 membranes by mito-VPS41 was greatly enhanced by the overexpression of Arl8b, whereas in cells knocked out for Arl8a-b, no recruitment was observed. Concomitantly, deletion of the Arl8-binding WD40 domain of VPS41 prevented recruitment of LAMP1 membranes. By immuno-EM, we showed that the LAMP1-positive membranes recruited by mito-VPS41 mainly consisted of a heterogeneous population of small vesicles, sized 50–200 nm. Strikingly, Arl8b was present on these vesicles, indicating a direct role in their transport and endosomal targeting. To further determine the identity of the Arl8b/LAMP1-positive vesicles, we used a combination of approaches to mark several different transport pathways. We found no LC3 on mitochondria, and by immuno-EM, Tom20 was absent from the recruited vesicles; hence, there was no sign of mitophagy (Onishi et al., 2021). Then, since a portion of LAMPs exits the TGN in AP1/clathrin-coated carriers for CI-M6PR and lysosomal enzymes, we investigated a possible redistribution of CI-M6PR. Unlike LAMP1, CI-M6PR did not redistribute to mitochondria, and by EM, we found that the recruited vesicles were not coated with clathrin. These data ruled out a role of VPS41 in CI-M6PR transport, which is consistent with the model that CI-M6PR enters the endo/lysosomal system at early endosomes that lack VPS41 (Waguri et al., 2003; Von Figura and Hasilik, 1986; Kornfeld and Mellman, 1989; Pols et al., 2013b). Furthermore, we found no redistribution of endocytosed dextran and internalized BSA^5^ was absent from the vesicles recruited by mito-VPS41, indicating that they were not derived from endocytic pathways. Finally, we combined the mito-VPS41 recruitment assay with the RUSH system to control and synchronize LAMP2A release from the ER. This showed that newly synthesized LAMP2A was indeed recruited by mito-VPS41. Recruitment was visible already 20 min after release of the ER block, which is consistent with ER–Golgi–TGN traveling kinetics of LAMP2A.

These combined assays demonstrated that most LAMP1/Arl8b membranes recruited by mito-VPS41 were LAMP carriers carrying newly synthesized LAMPs from the TGN. It remains possible that some vesicles seen by EM were of a different origin, but they would be a minority. By EM, we found some endo/lysosomes in the vicinity of mito-VPS41–covered mitochondria, but it was not evident whether these were tethered and fluorescence staining of cathepsin D (Fig. S5 E) or internalized dextran did not show an alteration in the overall distribution of endo/lysosomes. Notably, the mito-VPS41 recruitment studies were conducted in VPS41KO cells, which prevents formation of the HOPS complex and induces HOPS-dependent alterations in the endo/lysosomal system (van der Beek et al., 2024), such as the formation of hybrid early-late endosomes.

A major implication of the LAMP carrier pathway is that transport of lysosomal membrane proteins and M6PR/lysosomal enzymes can be independently regulated (Pols et al., 2013b), allowing the sequential delivery of distinct components to the maturing endosomal system. LAMP carriers have thus far been implicated in the transport of the lysosomal membrane proteins LAMP1, LAMP2, VAMP7, NPC1, and V0-ATPase (Pols et al., 2013b; Swetha et al., 2011), and it is likely that more lysosomal membrane proteins use this pathway. Targeted input of membrane proteins to late endosomes will result in increased local concentrations, which, e.g., explains the jump in LAMP levels in lysosomes and the prevention of premature acidification by V0-ATPase.

The presence and requirement of Arl8b on LAMP carriers was unexpected, since Arl8b is known as a regulator of transport and fusion events of endosomes and lysosomes. On endo/lysosomes, Arl8b recruits the effector protein SKIP (also known as PLEKHM2), which recruits kinesin-1 for binding to microtubules and anterograde transport (Rosa-Ferreira and Munro, 2011). In cooperation with the HOPS complex, SKIP recruits the GAP of Rab7, TBC1D15, permitting the switch from Rab7 to Arl8b (Jongsma et al., 2020). In addition, the Rab7-Arl8b effector PLEKHM1 is important for late endosome–lysosome fusion (Marwaha et al., 2017). Furthermore, a recent study suggested that Arl8b mediates retrograde movement of endo/lysosomes via interactions with the effectors RUFY3 and RUFY4 (Keren-Kaplan et al., 2022), and very recently, Arl8b via RUFY1 was implicated in retrograde endosome-to-TGN trafficking of CI-M6PR (Rawat et al., 2023). In the present study, we demonstrated that Arl8b is necessary on TGN-derived LAMP carriers to target them to the WD40 domain of VPS41.

In VPS41KO cells that did not express mito-VPS41, Arl8b/LAMP-positive carriers accumulated around endo/lysosomes, their natural destination. The distance between accumulated LAMP carriers and endo/lysosomes often exceeded 28–40 nm, the estimated size of the HOPS complex (Shvarev et al., 2022). This suggested that in Arl8b-expressing VPS41KO cells, LAMP carriers traveled via an Arl8b microtubule–dependent pathway to late endosomes–lysosomes, but then failed to tether or fuse, resulting in local accumulations. Together, our observations implied a thus far unknown role of Arl8b in biosynthetic trafficking and a new insight into the function of Arl8b in lysosomal biogenesis (Fig. 10 E). Interestingly, recent studies in neurons implicated Arl8b in the anterograde axonal transport of TGN-derived “lysosome-related vesicles” that by immunofluorescence contained LAMP1 and synaptic vesicle proteins (Vukoja et al., 2018; Klassen et al., 2010). Combined, our data suggest that Arl8b can function in specific TGN exits, both in non-polarized cells and in highly differentiated cells, such as neurons.

We showed that Arl8b on LAMP carriers was necessary for recruitment by VPS41, but vice versa, we found that Arl8b could also recruit VPS41 to endo/lysosomes, which is consistent with previous studies (Garg et al., 2011; Marwaha et al., 2017; Khatter et al., 2015a). The expression of mito-Arl8b in HeLa^Arl8a-bKO^ cells resulted in a partial redistribution of VPS41^WT^-V5 to mitochondria, confirming the proposed role of Arl8b in recruitment of VPS41 to endo/lysosomes. However, we also observed that low levels of VPS41 could be recruited to endo/lysosomes in the absence of Arl8b. Together, these data indicate that VPS41 and Arl8b can be recruited to endo/lysosomes independent of each other and that interactions between these two proteins—in different constellations—are important for various steps in endo/lysosomal biogenesis.

Previously, we also observed the localization of exogenous VPS41^WT^ on LAMP carriers (Pols et al., 2013b). However, the function of this pool remains unclear. Notably, our recruitment assays were performed in VPS41KO cells expressing solely mitochondrial-localized VPS41, implicating that recruitment does not require the presence of VPS41 on LAMP carriers. This, however, does not rule out that in physiological conditions, VPS41 on LAMP carriers contributes to tethering by binding to Arl8b present on endosomes. Alternatively, VPS41 may piggyride on LAMP carriers for targeted delivery to endosomes.

Our study highlights several distinctions between the ALP pathway in yeast and the LAMP carrier pathway in mammalian cells. First, the ALP pathway depends on the association between VPS41 and AP3 and recent studies using yeast-VPS41 ectopically relocalized to mitochondria indicated that VPS41 binds incoming AP3 vesicles (Schoppe et al., 2020). We expressed mito-VPS41 in PC12^VPS41KO^ cells, which contain higher levels of AP3 than HeLa cells allowing us to stain for the endogenous protein. However, although the AP3-VPS41 interaction is conserved in mammalian cells (Cabrera et al., 2010; Asensio et al., 2013), we found no effect of mito-VPS41 on the overall localization of AP3, implying that mammalian VPS41 did not recruit AP3 or AP3-positive vesicles (Schoppe et al., 2020). These contradicting data reflect the different roles proposed for AP3 in yeast and mammalian cells. In yeast, AP3 is required for transporting vacuolar membrane proteins from the Golgi to the vacuole (Cowles et al., 1997a; Darsow et al., 1998; Wen et al., 2007), whereas in mammalian cells, AP3 transports LAMPs from tubular sorting endosomes to late endo/lysosomes (Peden et al., 2004; Dell’Angelica et al., 1998; Theos et al., 2005). Second, Arl8b is absent in yeast, as are LAMPs (Hofmann and Munro, 2006). This emphasizes the importance of studying species-specific mechanisms that arise from a distinct organization of the endo/lysosomal system.

We found that the recruitment of LAMP carriers by mito-VPS41 did not require the presence of other HOPS subunits and that this recruitment property was exclusive to VPS41, not shared by other HOPS subunits. However, in physiological conditions, VPS41 functions on endo/lysosomal compartments where the HOPS complex is present. This raises the question whether endogenous VPS41 present on endosomes exerts LAMP carrier recruitment alone or while being part of the HOPS complex. Interestingly, recent structural studies have shown that the VPS41 WD40 domain remains open when assembled within the HOPS complex, predicting that HOPS-associated VPS41 can still bind Arl8b (Shvarev et al., 2022; Khatter et al., 2015a). In agreement herewith, we found that the co-expression of mito-VPS41 with VPS18 did not lower the level of LAMP recruitment to mitochondria, indicating that binding to the VPS41 RING domain (VPS18) and WD40 domain (Arl8b) is not competitive. Furthermore, although mito-VPS18 was not able to recruit LAMP carriers, we found in a parallel study that KO of VPS18 or other HOPS subunits results in a similar phenotype (lack of endosomal maturation) in HeLa cells, indicating that likely all of HOPS is required for the fusion of the LAMP carriers (van der Beek et al., 2024) with endo/lysosomes. It is therefore tempting to speculate that VPS41 as part of the HOPS complex is the essential subunit for recruitment of LAMP carriers, while HOPS is required to mediate their subsequent fusion with endo/lysosomes (Fig. 10 E).

In summary, our investigations present evidence for the ability of mammalian VPS41 to recruit LAMP carriers, a function distinct from other HOPS subunits. Furthermore, we unveil an unexpected role of Arl8b in the LAMP carrier pathway, expanding our understanding beyond the conventional role of VPS41–Arl8b interaction in endosome–lysosome fusions. We propose that the presence of VPS41 on endosomal membranes is required to tether incoming LAMP carriers by binding to Arl8b (Fig. 10 E), whereas subsequent fusion of LAMP carriers with endosomes is dependent on the entire HOPS complex, as is fusion of endosomes with lysosomes. In this scenario, VPS41 as part of the HOPS complex defines a crucial step in cargo transfer from late endosomes and autophagosomes to lysosomes, and within the HOPS complex, VPS41 is required for recruitment of carriers transporting newly synthesized lysosomal membrane proteins. By integrating these two functions, VPS41 fulfills a unique role within the HOPS complex to enable lysosome biogenesis. Future investigations utilizing unbiased proteomics techniques may uncover additional cargo proteins for the LAMP carrier pathway, as well as reveal additional binding partners of VPS41, offering further insight into how VPS41 executes its multiple tasks.

## Materials and methods

### Cell culture and treatments

HeLa^VPS18KO^, HeLa^VPS39KO^, and HeLa^VPS41KO^ cell lines were made as described previously (van der Welle et al., 2021). HeLa^Arl8a-b^ CRISPR/Cas9 double-KO cells were kindly provided by Juan Bonifacino (National Institutes of Health, Bethesda, MD, USA) and previously described in Keren-Kaplan et al. (2022). HeLa^GFP-ARL8b KI^ were kindly provided by Dr. Jacques Neefjes (Leiden University Medical Center [LUMC], Leiden, Netherlands) and previously used in Jongsma et al. (2020). All HeLa cells were cultured in High-Glucose DMEM (Invitrogen) supplemented with 10% FBS (Bodinco BV) and 1% penicillin–streptomycin (cat no. P0781; Sigma-Aldrich) in a 5% CO_2_-humidified incubator at 37°C. PC12^VPS41KO^ cells were kindly provided by Cedric Asensio, Department of Biological Sciences at the University of Denver. PC12 cells were cultured in High-Glucose DMEM (Invitrogen) supplemented with 5% FBS, 10% horse serum, and 1% penicillin–streptomycin (cat no. P0781; Sigma-Aldrich) in a 5% CO_2_-humidified incubator at 37°C. HeLa^VPS41KO^ cells stably expressing mito-Cherry-FRB were made from HeLa^VPS41KO^ cells transfected with pMinitol2-hEF1α-MCS-polyA-pgk-Puro (empty vector for generating stable cell lines via the transposase system) + mito-Cherry-FRB (pMito-mCherry-FRB was a gift from Stephen Royle (University of Warwick, Coventry, UK) (plasmid #59352; Addgene; http://n2t.net/addgene:59352; RRID:Addgene_59352) using the PiggyBac transposon system.

For fluorescence microscopy, X-tremeGENE HP (cat no. 6366546001; Roche) was used for transfection according to the manufacturer’s instructions, unless otherwise indicated. Briefly, cells were incubated with 0.5 μg plasmid with 0.5 ml of transfection ratio 1:1 overnight for transfection in a 24-well plate. For EM, cells were grown in 6-cm petri dishes and transfected with Effectene (cat no. 301427; Qiagen) according to the manufacturer’s instructions.

To study the endocytic pathway, cells expressing mito-VPS41 were incubated with a medium containing dextran–Alexa Fluor 568 (ratio 1:100, cat no. D22912; Invitrogen) for 2 h. After five washings with PBS at 37°C, cells were fixed and embedded as described in the Fluorescence microscopy section.

To study recruitment of acidic endo/lysosomal compartments, we prepared two sets of HeLa^VPS41KO^ cells co-expressing mito-VPS41-GFP and Arl8b-HA. One set was treated with LysoTracker dye (1:10,000, cat no. L12492; Invitrogen) for 30 min, followed by five washes with 37°C PBS, fixation with 4% PFA, and embedding in ProLong-DAPI, and imaged. The other set was fixed with 4% PFA, permeabilized with Triton, and stained for Arl8b-HA. Although PFA fixation caused slight LysoTracker leakage and increased cytoplasmic background, it was necessary to assess Arl8b-HA expression levels through immunostaining in parallel samples (>90% of the cells, Fig. S3 F). KD of Arl8b was achieved using On-Target siGENOME SMARTpool of Arl8b siRNAs (D-020294-01 #5′-GAU​GAG​AAA​CAG​CUA​AUU​G-3′; D-020294-02 #5′-CGA​AAU​GAG​CUA​CAU​AAU​C-3′; D-020294-03 #5′-GGU​AAC​GUC​ACA​AUA​AAG​A-3′; D-020294-04 #5′-GGA​CAA​CCC​CGA​UUU​CGA​A-3′). As a control, we used All-Stars negative control siRNA (cat no. 1027281; Qiagen). siRNA treatments were performed using HiPerFect transfection reagent (cat no. 301705; Qiagen) according to the manufacturer’s protocol.

### Fluorescence microscopy

Cells were washed twice with PBS and fixed with 4% wt/vol PFA (Polysciences, Inc.) for 30 min. Fixed cells were washed three times with PBS and permeabilized with 0.1% Triton X-100 (Sigma-Aldrich) for 10 min. After permeabilization, cells were incubated with 1% BSA solution for 15 min (blocking buffer). Cells were then incubated with the primary antibody diluted in PBS/1% BSA for 1 h, washed with PBS, and subsequently incubated for 30 min with appropriate Alexa 488/568/647–conjugated secondary antibodies (Invitrogen) diluted 1:350 in PBS/1% BSA for 30 min. After three washings with PBS and one washing with DEMI water, coverslips were mounted on slides using ProLong-DAPI (cat no. P36971; Invitrogen). Images were taken on a Zeiss LSM700 confocal microscope fitted with a 63 × 1.4-NA oil-immersion Apochromat lens. All images presented are single z-planes. Unmodified single focal plane images were quantified using Fiji. Briefly, to identify the proteins’ recruitment on mitochondria, we performed the following steps. First, we segmented the regions of mitochondria based on the signal of the mito-tagged protein. Second, we adjusted the signal threshold of mitochondria and created a mask. Third, we selected cells that expressed both the mito-tagged protein and the protein of interest and isolated only the cells that were positive for the protein of interest. Fourth, we calculated the mean intensity of the protein of interest on the mitochondrial segment and normalized this value to fold change for the control. In each experiment, we normalized the degree of overlap to diffusely distributed control proteins, such as GFP, and set that control condition to 1. All other data in the graphs refer to the number of times the signal exceeded this basal level. For Pearson’s correlation, we used Volocity 6.3 software. For live-cell experiments, cells were seeded on 15-µ-slide 8-well ibidi glass coverslips (cat no. 80827). Fluorescence imaging was performed in a 5% CO_2_-humidified incubator at 37°C on a Leica Thunder fluorescence microscope with a 100×, 1.47-NA oil objective, a Photometrics Prime 95B scientific CMOS camera, and LAS X software.

### Statistical analysis

Each experiment was independently replicated a minimum of three times (green dots in superplots represent the mean of each replicate), grey dots represent individual cell data point. Results are presented as the mean ± SEM from at least three independent experiments. Statistical significance was assessed using Prism 9 (GraphPad), with detailed methods provided in the figure legends.

### Immuno-electron microscopy

Preparation of cells for cryosections and immunogold labeling was performed as described in Slot and Geuze (2007). In brief, cells were fixed by adding freshly prepared solution of either 4% PFA or 4% PFA, 0.4% GA in 0.1 M phosphate buffer (pH 7.4) (2× solution) to an equal volume of culture medium. After 5 min of incubation, the fixative was replaced with either 4% PFA or 2% PFA, 0.2% GA in 0.1 M phosphate buffer (pH 7.4) (1× solution) and fixed for 2 h at RT before storage at 1% PFA at 4°C until further processing. Next, cells were washed with PBS/0.05 M glycine, scraped in 1% gelatin in PBS, and pelleted in 12% gelatin in PBS. Cell pellets were then solidified on ice, cut into blocks, infiltrated overnight in 2.3 M sucrose, mounted on aluminum pins, and stored in liquid nitrogen. 60 to 80 nm ultrathin sections were obtained using a UC7 ultra-cryomicrotome (Leica) and collected on a grid using a 1:1 mixture of 2.3 M and 1.8% wt/vol methylcellulose. Images were obtained on a JEOL TEM-1011 electron microscope equipped with a Veleta 2kx2k CCD camera (EMSIS).

### Antibodies

Antibodies used in this study are specified in Table 1.

**Table 1:** Antibodies used

Primary antibody	Use	Dilution	Supplier	Cat no.
Tom20	IF	1:400	BD Transduction Laboratories	612278
Tom20	EM	1:100	BD Transduction Laboratories	612278
V5 R	IF	1:400	Sigma-Aldrich	V8137
V5 R	EM	1:500	Sigma-Aldrich	V8137
V5 M	IF	1:200	Invitrogen	R96025
V5 M	EM	1:150	Invitrogen	R96025
LAMP1 CD107a M	IF	1:500	BD Pharmingen	555798
LAMP1 CD107a M	EM	1:150	BD Pharmingen	555798
LAMP1 (D2D11) R	EM	1:100	Cell signaling	9091
VPS18 R	IF	1:200	Abcam	ab178416
Flag TM R	IF	1:200	Sigma-Aldrich	F7425
LC3-1703 M	IF	1:200	Cosmo Bio Co. LTD	CTB-LC3-2-IC
CI-M6PR R	IF	1:500	Gift from G. Lienhard^a^	
HA 11 COVANCE M	IF	1:300	BioLegend	901502
HA 11 COVANCE M	EM	1:300	BioLegend	901502
HA R	IF	1:300	Sigma-Aldrich	H6908
ARL8A/B (H-8) M	WB	1:1,000	Santa Cruz	sc398635
ARL8B R	WB	1:1,000	Proteintech	13049-1-ap
GFP G	EM	1:300	Rockland	600-106-215
Actin M	WB	1:5,000	MP Biomedicals	69100
LAMP2A M	EM	1:150	BD Pharmingen	555803
LAMP2A M	IF	1:400	BD Pharmingen	555803
AP3D1 M	IF	1:200	DSHB	AB_2056641
VPS41 M	WB	1:1,000	Abcam	ab181078
CatD M	IF	1:500	RD Systems	AF953
Secondary antibody	Use	Dilution	Supplier	Cat no.
DaM–Alexa 488	IF	1:350	Thermo Fisher Scientific	A21202
DaR–Alexa 568	IF	1:350	Thermo Fisher Scientific	A10042
GaM–Alexa 647	IF	1:350	Thermo Fisher Scientific	A21235
GaR–Alexa 568	IF	1:350	Thermo Fisher Scientific	A11036
ChaR–Alexa 647	IF	1:350	Thermo Fisher Scientific	A21443
DaG–Alexa 647	IF	1:350	Thermo Fisher Scientific	A21447
GaM–Alexa 680	WB	1:10,000	Thermo Fisher Scientific	A21057
GaR–IRDye 800	WB	1:10,000	LI-COR	926-32211
RaM–IgG	EM	1:200	Rockland	610-4120

M, mouse; R, rabbit; D, donkey; G, goat; Ch, chicken.

a G. Lienhard (Dartmouth Medical School, Hanover, NH, USA) used in the paper Klumperman et al. (1993).

### Western blotting

Cells were washed twice with ice-cold PBS and incubated for 15 min with lysis buffer containing CHAPS 1% (50 mM Tris, pH = 7.5, 150 mM NaCl, 5 mM MgCl_2_, 1 mM DTT, 1% [wt/vol] CHAPS) supplemented with protease and phosphatase inhibitor (cat no. 5056489001 and 4906837001; Roche). Cells were scraped, and lysates were centrifuged at 13,200 rpm at 4°C. Supernatants were collected, and protein levels were equalized using Bradford Protein Assay (cat no. 5000006; Bio-Rad). Samples were then eluted with SDS sample buffer and boiled for 5 min at 95°C. Samples were loaded and run on Mini-PROTEAN TGX precast gels (4–15%) (cat no. 456-1086; Bio-Rad). Proteins were transferred to PVDF membranes (cat no. 1704272; Bio-Rad) using the Trans-Blot Turbo system (Bio-Rad). Membranes were blocked with Intercept (TBS) Blocking Buffer (cat no. 927-60001; LI-COR) for 1 h at RT. Afterward, membranes were incubated with the primary antibody in Intercept (TBS) Blocking Buffer overnight at 4°C. After extensive washings with TBS/0.1% Tween, membranes were incubated with secondary antibody in Intercept (TBS) Blocking Buffer for 30 min. After another washing with TBS/0.1% Tween, membranes were imaged using Amersham Typhoon Laser Scanner (GE Healthcare Life Sciences).

### Plasmids

To generate plasmids expressing mito-VPS41^WT^-V5, we PCR-ed the N-terminal mitochondrial targeting sequence of Tom70 (GenBank accession number AAI39422.1) from the pENTR Tom70-GFP plasmid kindly provided by Alistair Hume (University of Nottingham, Nottingham, UK) using primers Fw = (5′-3′) ACA​CGG​TAC​CAT​GGC​CGC​CTC​TAA​GCC​CAT​AG and Rv = (3′-5′) ACA​CGG​TAC​CCA​GGC​CGC​TGG​CGT​CGC​CCC​GGC. The PCR segment was cut with the restriction enzyme KpnI and pasted into VPS41^WT^-APEX2-V5 plasmids previously used in our laboratory (van der Welle et al., 2021). The APEX sequence, which can be used for potential future proximity labeling experiments, does not affect the function of VPS41 as was previously shown by rescue experiments in VPS41KO cells (van der Welle et al., 2021). The same approach was used to obtain mito-VPS41^R662*^-V5 and mito-V5 with as destination vectors VPS41^R662*^-APEX2-V5 and EV-APEX2-V5, also previously used in van der Welle et al. (2021). VPS18-GFP, mCherry-VPS11, and VPS18-Flag were kindly provided by Rik van der Kant (VU Amsterdam, Free University of Amsterdam); to generate mito-VPS18-GFP, we PCR-ed VPS18 from a VPS18-GFP plasmid (Fw = [5′-3′] ACA​CAA​GCT​TAT​GGC​GTC​CAT​CCT​GGA​TG; Rv = [3′-5′] CAC​GGA​TCC​AAC​AGC​CAA​CTG​AGC​TGC​TCC​TC); then, we inserted the amplified segment to a pENTR Tom70-GFP plasmid using restriction sites HindIII and BamHI. A similar approach was used to generate mito-VPS39-GFP. Restriction sites used were KpnI and BamHI (primers used Fw = [5′-3′] AGA​GGG​TAC​CAA​TGC​ACG​ACG​CTT​TCG​AGC; Rv = [3′-5′] AGA​GGG​ATC​CGT​GTC​AGC​TGG​GTT​TAC​CTC). Rab7-GFP and RILP-GFP were kindly provided by Jacques Neefjes (LUMC, Leiden, Netherlands). pDEST47-ARL8B was a gift from Richard Kahn (Emory University School of Medicine, Atlanta, GA, USA) (plasmid #67467; Addgene; https://n2t.net/addgene:67467; RRID:Addgene_67467); then, the Arl8b segment was amplified using primers Fw = (5′-3′) GGG​GAC​AAG​TTT​GTA​CAA​AAA​AGC​AGG​CTT​AAT​GCT​GGC​GCT​CAT​CTC​C; Rv=(3′-5′) GGG​GAC​CAC​TTT​GTA​CAA​GAA​AGC​TGG​GTT​GCT​TCT​TCT​AGA​TTT​TGA​ATG​CTG​AAT​AAG​CC) and cloned into a pDONR201 vector via a Gateway BP Clonase II Enzyme mix (cat no. 10348102; Invitrogen). Afterward, Arl8b-GFP and Arl8b-HA were obtained using a Gateway LR Clonase II Enzyme mix (cat no. 10134992; Invitrogen), using destination vectors pDest-eGFP-N1, which was a gift from R. Shaw(University of Utah. Salt Lake City, UT, USA) (plasmid #31796; Addgene; https://n2t.net/addgene:31796; RRID:Addgene_31796), and pDest520 (4.2), which was a gift from Ronald Roepman (Radboud University, Nijmegen, Netherlands). To obtain GFP-Rab2, we amplified the GTPases from myc-Rab2 (gift from T. Hébert [McGill University, Montréal, Canada] [plasmid #46779; Addgene; https://n2t.net/addgene:46779; RRID:Addgene_46779]) using primers (Fw = [5′-3′] AGA​GGA​GCT​CAA​ATG​GCG​TAC​GCC​TAT​CTC​TTC; Rv = [3′-5′] AGA​GGA​ATT​CTC​AAC​AGC​AGC​CGC​CCC​CAG) and inserted the segment to pEGFP-C1 using SacI and EcoRI as restriction sites. Rab6-GFP was kindly provided by Ilya Grigoriev (University of Utrecht, Utrecht, Netherlands). Mito-VPS41^WT^-GFP and mito-VPS41^∆WD40^-GFP were generated by amplification of VPS41^WT^ WT Fw = (5′-3′) AGA​GGG​ATC​CCA​TGG​CGG​AAG​CAG​AGG​AGC​AG; Rv = (3′-5′) AGA​GGG​ATC​CAA​TTT​TTT​CAT​CTC​CAA​AAT​TGC or ∆WD40 Fw = (5′-3′) AGA​GGG​ATC​CAA​TGA​GGG​ATT​TGC​CAA​GTC​G; Rv = (3′-5′) AGA​GGG​ATC​CAA​TTT​TTT​CAT​CTC​CAA​AAT​TGC; and inserted to a pENTR Tom70-GFP plasmid using the restriction site BamHI. Mito-Arl8b-GFP was generated by PCR of the GTPase using primers Fw = (5′-3′) ACA​CGG​TAC​CAA​TGC​TGG​CGC​TCA​TCT​CCC​GC; Rv = (3′-5′) AGA​GGG​ATC​CAA​GCT​TCT​TCT​AGA​TTT​TGA​ATG; the segment was inserted to pENTR Tom70-GFP using KpnI and BamHI. LAMP2A-RUSH was kindly provided by Dr. Santiago Di Pietro (Colorado State University, Fort Collins, CO, USA). CD-M6PR-GFP-RUSH was a kind gift from Dr. Juan Bonifacino (National Institutes of Health, Bethesda, MD, USA) (plasmid #202797; Addgene; https://n2t.net/addgene:202797; RRID:Addgene_202797).

### RUSH assay

We used a bicistronic vector for LAMP2A fused with SBP and mNeonGreen or GFP (LAMP2A-RUSH) and combined this with streptavidin fused to the ER-specific KDEL sequence. HeLa^VPS41KO^ or HeLa^VPS18KO^ cells were seeded in full DMEM and transfected at 70–80% confluency with the plasmids mito-VPS41^WT^-V5 or mito-V5, SBP-LAMP2A-mNeonGreen, and Arl8b-HA. 1.5 μl of Lipofectamine 2000 (Thermo Fisher Scientific) was mixed with DNA (0.2–0.7 μg per construct/well) in 50 μl Opti-MEM (Thermo Fisher Scientific) and incubated for 20 min at RT, added to the cells, and incubated for 2–3 h at 37°C. The transfection mix was then removed and replaced for full medium. The following day, 100 μM of D-biotin (B4501; Sigma-Aldrich) was added to the medium, followed by incubation at 37°C for 1.5 h to induce the release of SBP-LAMP2A-NeonGreen from the KDEL–streptavidin hook. Cells were fixed in 4% PFA and immunolabeled for HA and V5. For simultaneous recruitment of VPS41 to mitochondria and ER release of LAMP2A, we transfected HeLa^VPS41KO^ stably expressing mito-mCherry-FRB with FKBP-VPS41^WT^-Halo and SBP-LAMP2A-GFP using Effectene. The next day, we performed live imaging in a 5% CO_2_-humidified incubator at 37°C without treatment (T0), followed by adding A/C heterodimerizer (rapalog 1:1,000, cat no. 635057; Takara) and biotin 40 μM (cat no. B4501; Sigma-Aldrich) using a Leica Thunder fluorescence microscope with a 100× 1.47-NA oil objective, a Photometrics Prime 95B scientific CMOS camera, and LAS X software. For quantification, we transfected HeLa^VPS41KO^ stably expressing mito-mCherry-FRB with FKBP-VPS41^WT^-Halo and LAMP2A-RUSH using Effectene. The day after, we treated the cells with A/C heterodimerizer (rapalog 1:1,000, cat no. 635057; Takara) and biotin 40 µM (cat no. B4501; Sigma-Aldrich). Then, we fixed the cells in 4% PFA after 45 min. After three washes with PBS and one wash with DEMI water, coverslips were mounted on slides using ProLong-DAPI (cat no. P36971; Invitrogen).

### Online supplemental material

Fig. S1 shows properties of VPS18 and VPS39 to recruit other HOPS subunits to ectopic locations. Fig. S2 shows the role of Arl8b in recruitment of LAMP1 and LAMP2A to mito-VPS41. Fig. S3 shows non-pseudocolored images of F6, F7, F8, and F9. Fig. S4 shows the time-lapse frames of FKBP-VPS41^WT^ recruitment to mito-FRB. Additionally, it displays the video frames of LAMP2A-RUSH vesicles to mito-VPS41^WT^ and control. Fig. S5 shows the accumulation of LAMP2-RUSH vesicles on mitochondria bearing FKBP-VPS41^WT^ upon biotin–rapalog treatment. Videos 1 and 2 show Golgi accumulation of LAMP2A-RUSH upon biotin treatment. Video 3 shows LAMP2A-RUSH biosynthetic vesicle accumulation to mitochondria harboring VPS41. Video 4 shows mitochondrial recruitment of FKBP-VPS41^WT^. Video 5 shows the absence of LAMP2A-RUSH vesicle accumulation in cells lacking VPS41 in the mitochondria. Video 6 shows the absence of FKBP-VPS41^WT^ recruitment to mito-FRB in cells not treated with rapalog. Data S1 shows source data for Figs. 1, 2, 3, 4, 5, 9, and 10 and supplementary figures.

## Supplementary Material

Data S1 shows source data for Figs. 1, 2, 3, 4, 5, 9, and 10 and supplementary figures.

SourceData FS2 is the source file for Fig. S2.

## Data Availability

Data can be obtained from the corresponding author upon reasonable request.
