# Supplementary figures and images for: VPS41 recruits biosynthetic LAMP-positive vesicles through interaction with Arl8b
Source: J Cell Biol. 2025 Feb 5;224(4):e202405002. doi: 10.1083/jcb.202405002 (PMC11809577; doi:10.1083/jcb.202405002)

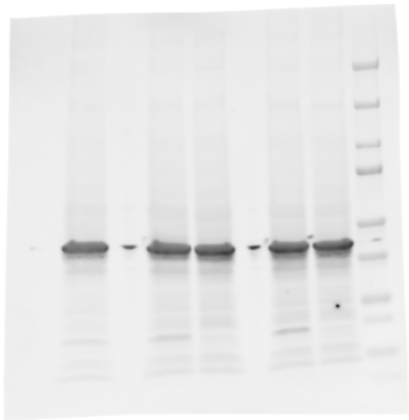

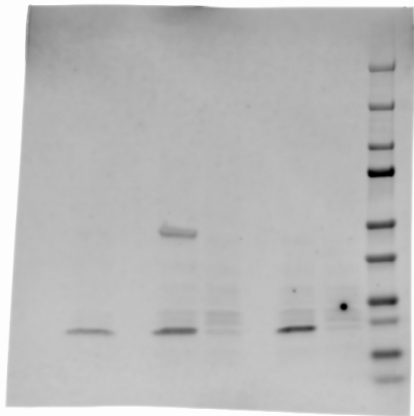

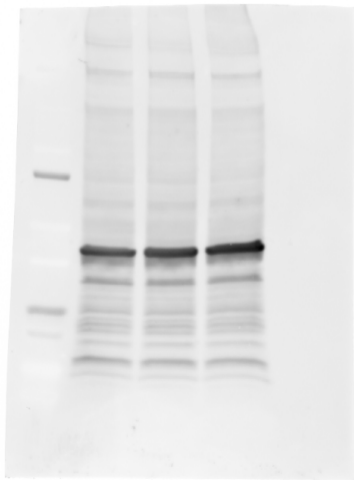

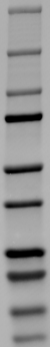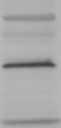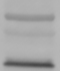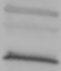

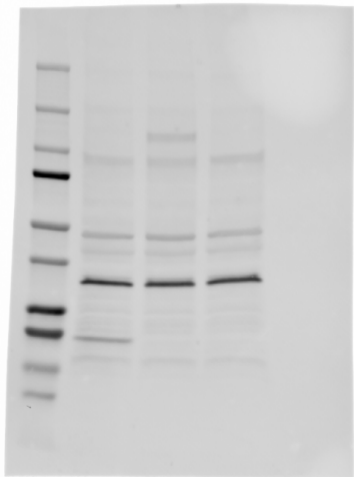

Supplement: SourceData FS2 — is the source file for Fig. S2. [file jcb_202405002_sourcedatafs2.pdf]
